# Supplementary material for: Base-Catalyzed Nucleophilic Addition Reaction of Indoles with Vinylene Carbonate: An Approach to Synthesize 4-Indolyl-1,3-dioxolanones
Source: Molecules. 2023 Nov 6;28(21):7450. doi: 10.3390/molecules28217450 (PMC10649246; doi:10.3390/molecules28217450)

Supporting Information

Base-Catalyzed Nucleophilic Addition Reaction of Indoles with Vinylene Carbonate: An Approach to Synthesize 4-Indolyl-1,3-dioxolanones

Xia Chen,[†,‡] Xiao-Yu Zhou,*[,†] and Ming Bao*[,‡]

[†]School of Chemistry and Materials Engineering, Liupanshui Normal University, Liupanshui 553004, China

[‡]State Key Laboratory of Fine Chemicals, Dalian University of Technology, Dalian 116024, China

Table of Contents

1. General and Materials……………………………………………………………………S3

2. The Typical Procedure for the Addition of Indoles with Vinylene Carbonate……..….S3-10

3. Substrate Extension Studies………………………………………………………………S10-11

4. X-Ray Crystallographic Analysis.………………………………………..…………..S11-13

5. Copy of NMR for the Products…………………………………..…....………..….S14-40

1. General Information.

Unless otherwise noted, all reactions were carried out in oven-dried 25-mL Schlenk tubes under a nitrogen atmosphere. IKA plate was used as the heat source. All reagents and solvents were of pure analytical grade. Thin layer chromatography (TLC) was performed on HSGF254 silica gel, pre-coated on glass-backed plates coated with 0.2 mm silica and revealed with either a UV lamp (λ_{max} = 254 nm). The products were purified by flash column chromatography on silica gel 200-300 mesh. ^{1}H and ^{13}C NMR spectra were recorded on a 400 MHz spectrometer (^{1}H 400 MHz, ^{13}C 101 MHz) using d_6-DMSO or $CDCl_3$ as the solvent with tetramethylsilane (TMS) as the internal standard at room temperature. The chemical shifts are reported in ppm downfield (δ) from TMS, the coupling constants J are given in Hz. The peak patterns are indicated as follows: s, singlet; d, doublet; t, triplet; q, quartet; m, multiplet. High resolution mass spectra were recorded on either a Q-TOF mass spectrometry or a LTQ Orbitrap XL mass spectrometry. X-ray crystallography analysis was performed on a Bruker D8 Quest X-ray diffractionmeter.

2. The Typical Procedure for Nucleophilic Addition between Indoles with Vinylene Carbonate

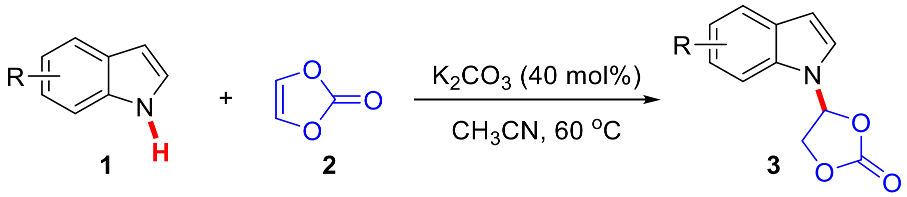

A mixture of indoles **1** (0.50 mmol), vinylene carbonate (172 mg, 2.0 mmol, 4.0 equiv) and K_2CO_3 (27.6 mg, 0.20 mmol, 40 mol%) in CH_3CN (3 mL) was added into a Schlenk flask (25 mL) and stirred at 60 °C. After the reaction was finished, the solvent was evaporated under reduced pressure and the residue was purified by column chromatography (petroleum ether/ethyl acetate 5:1 to 1:1) to provide the product **3**.

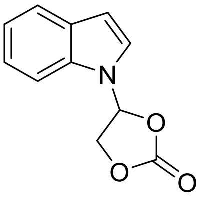

4-(1*H*-indol-1-yl)-1,3-dioxolan-2-one (3a): Yield: 80%, 80.9 mg, white solid, mp 132-134 °C, R_f = 0.41 (H/E = 2:1). ^{1}H NMR (400 MHz, d_6-DMSO) δ 7.71 (d, J = 3.4 Hz, 1H), 7.64 (d, J = 7.8 Hz, 1H), 7.59 (d, J = 8.2 Hz, 1H), 7.28 (t, J = 7.7 Hz, 1H), 7.23 (t, J = 6.3 Hz, 1H), 7.18 (t, J = 7.5 Hz, 1H), 6.69 (d, J = 3.3 Hz, 1H), 5.04 (d, J = 6.3 Hz, 2H). ^{13}C NMR (101 MHz, d_6-DMSO) δ 154.1, 136.0, 129.5, 126.2, 123.2, 121.6, 121.6, 110.4, 105.6, 82.3, 68.4. $[M + H]^+$ calcd for $C_{11}H_{10}NO_3$, 204.0661; found 204.0657.

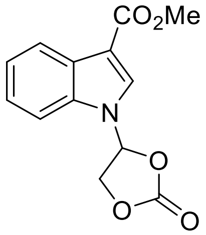

methyl 1-(2-oxo-1,3-dioxolan-4-yl)-1*H*-indole-3-carboxylate (3e): Yield: 66%, 86.6 mg, white solid, mp 206-208 °C, R_f = 0.32 (H/E = 2:1). ^{1}H NMR (400 MHz, d_6-DMSO) δ 8.53 (s, 1H), 8.10 (d, J = 7.8 Hz, 1H), 7.63 (d, J = 8.1 Hz, 1H), 7.42-7.33 (m, 2H), 7.28-7.21 (m, 1H), 5.17-4.99 (m, 2H), 3.86 (s, 3H). ^{13}C NMR (101 MHz, d_6-DMSO) δ 164.5, 153.8, 136.0, 133.5, 126.9, 124.4, 123.5, 121.7, 111.2, 109.5, 82.4, 68.3, 51.6. HRMS (ESI) m/z: $[M + H]^+$ calcd for $C_{13}H_{12}NO_5$, 262.0715; found 262.0714.

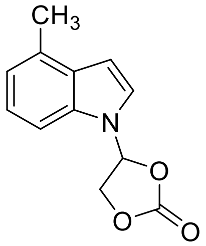

4-(4-methyl-1*H*-indol-1-yl)-1,3-dioxolan-2-one (3f): Yield: 53%, 57.7 mg, white solid, mp 118-120 °C, R_f = 0.36 (H/E = 2:1). ^{1}H NMR (400 MHz, d_6-DMSO) δ 7.68 (d, J = 3.4 Hz, 1H), 7.39 (d, J = 8.2 Hz, 1H), 7.23-7.14 (m, 2H), 6.98 (d, J = 7.2 Hz, 1H), 6.72 (d, J = 3.3 Hz, 1H), 5.03 (d, J = 6.4 Hz, 2H), 2.49 (s, 3H). ^{13}C NMR (101 MHz, d_6-DMSO) δ 154.1, 135.7, 130.6, 129.4, 125.6, 123.3, 121.7, 107.9, 104.1, 82.5, 68.4, 18.7. HRMS (ESI) m/z: $[M + H]^+$ calcd for $C_{12}H_{12}NO_3$, 218.0817; found 218.0815.

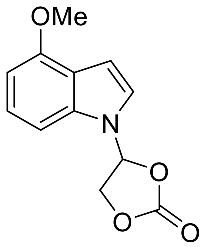

4-(4-methoxy-1*H*-indol-1-yl)-1,3-dioxolan-2-one (3g): Yield: 74%, 86.0 mg, white solid, mp 172-174 °C, R_f = 0.33 (H/E = 2:1). ^{1}H NMR (400 MHz, d_6-DMSO) δ 7.59 (d, J = 3.4 Hz, 1H), 7.23-7.15 (m, 3H), 6.69 (d, J = 7.6 Hz, 1H), 6.66 (d, J = 3.2 Hz, 1H), 5.01 (d, J = 6.4 Hz, 2H), 3.89 (s, 3H). ^{13}C NMR (101 MHz, d_6-DMSO) δ 154.0, 153.4, 137.3, 124.7, 124.4, 119.7, 103.5, 102.6, 101.9, 82.5, 68.4, 55.6. HRMS (ESI) m/z: $[M + H]^+$ calcd for $C_{12}H_{12}NO_4$, 234.0766; found 234.0765.

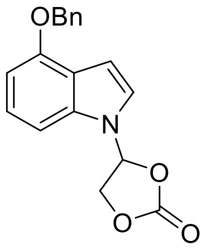

4-(4-(benzyloxy)-1*H*-indol-1-yl)-1,3-dioxolan-2-one (3h): Yield: 65%, 100.3 mg, white solid, mp 146-148 °C, R_f = 0.32 (H/E = 2:1). ^{1}H NMR (400 MHz, d_6-DMSO) δ 7.61 (d, J = 3.4 Hz, 1H), 7.51 (d, J =

7.6 Hz, 2H), 7.41 (t, J = 7.4 Hz, 2H), 7.34 (t, J = 7.1 Hz, 1H), 7.21-7.14 (m, 3H), 6.78 (d, J = 6.5 Hz, 1H), 6.71 (d, J = 3.3 Hz, 1H), 5.26 (s, 2H), 5.02 (d, J = 6.4 Hz, 2H). ^{13}C NMR (101 MHz, d_6-DMSO) δ 154.0, 152.4, 137.8, 137.4, 128.9, 128.2, 127.9, 124.9, 124.3, 120.1, 103.7, 103.4, 102.6, 82.5, 69.6, 68.4. HRMS (ESI) m/z: $[M + H]^+$ calcd for $C_{18}H_{16}NO_4$, 310.1079; found 310.1075.

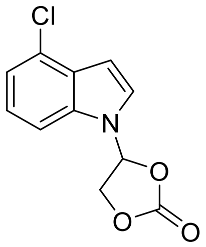

4-(4-chloro-1*H*-indol-1-yl)-1,3-dioxolan-2-one (3i): Yield: 84%, 100.0 mg, white solid, mp 130-132 °C, R_f = 0.48 (H/E = 2:1). ^{1}H NMR (400 MHz, d_6-DMSO) δ 7.86 (d, J = 3.5 Hz, 1H), 7.59 (d, J = 7.6 Hz, 1H), 7.33-7.22 (m, 3H), 6.73 (d, J = 3.3 Hz, 1H), 5.05 (d, J = 6.3 Hz, 2H). ^{13}C NMR (101 MHz, d_6-DMSO) δ 153.9, 136.8, 127.8, 127.5, 125.5, 124.2, 121.2, 109.7, 103.4, 82.3, 68.5. HRMS (ESI) m/z: $[M + H]^+$ calcd for $C_{11}H_9ClNO_3$, 238.0271; found 238.0266.

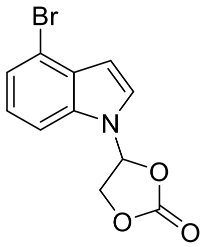

4-(4-bromo-1*H*-indol-1-yl)-1,3-dioxolan-2-one (3j): Yield: 87%, 123.0 mg, white solid, mp 154-156 °C, R_f = 0.36 (H/E = 2:1). ^{1}H NMR (400 MHz, d_6-DMSO) δ 7.87 (d, J = 3.4 Hz, 1H), 7.64 (d, J = 8.3 Hz, 1H), 7.41 (d, J = 7.6 Hz, 1H), 7.23 (t, J = 7.3 Hz, 2H), 6.65 (d, J = 3.4 Hz, 1H), 5.05 (d, J = 6.3 Hz, 2H). ^{13}C NMR (101 MHz, d_6-DMSO) δ 153.9, 136.4, 133.2, 129.7, 127.5, 124.5, 124.3, 114.5, 110.2, 105.1, 82.3, 68.5. HRMS (ESI) m/z: $[M + H]^+$ calcd for $C_{11}H_9BrNO_3$, 281.9766; found 281.9758.

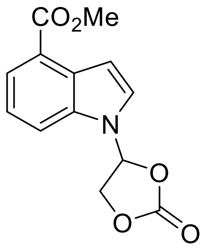

methyl 1-(2-oxo-1,3-dioxolan-4-yl)-1*H*-indole-4-carboxylate (3k): Yield: 91%, 119.0 mg, white solid, mp 146-148 °C, R_f = 0.39 (H/E = 2:1). ^{1}H NMR (400 MHz, d_6-DMSO) δ 7.92 (d, J = 7.5 Hz, 2H), 7.88 (d, J = 7.5 Hz, 1H), 7.41 (t, J = 7.9 Hz, 1H), 7.30 (t, J = 6.2 Hz, 1H), 7.19 (d, J = 3.3 Hz, 1H), 5.07 (dd, J = 8.6, 3.5 Hz, 2H), 3.92 (s, 3H). ^{13}C NMR (101 MHz, d_6-DMSO) δ 167.1, 153.9, 136.9, 128.8, 128.3, 124.5, 122.7, 121.8, 115.6, 106.2, 82.0, 68.5, 52.4. HRMS (ESI) m/z: $[M + H]^+$ calcd for $C_{13}H_{12}NO_5$, 262.0715; found 262.0708.

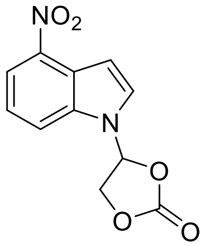

4-(4-nitro-1*H*-indol-1-yl)-1,3-dioxolan-2-one (3l): Yield: 90%, 111.7 mg, light yellow solid, mp 188-190 °C, R_f = 0.30 (H/E = 2:1). ^{1}H NMR (400 MHz, d_6-DMSO) δ 8.20 (d, J = 8.0 Hz, 1H), 8.15 (d, J = 8.5 Hz, 2H), 7.53 (t, J = 8.1 Hz, 1H), 7.36 (t, J = 6.2 Hz, 1H), 7.26 (d, J = 3.3 Hz, 1H), 5.13 – 5.04 (m, 2H). ^{13}C NMR (101 MHz, d_6-DMSO) δ 153.8, 140.2, 138.2, 131.0, 123.1, 122.8, 119.0, 118.1, 104.8, 81.9, 68.67. HRMS (ESI) m/z: [M + H]$^+$ calcd for $C_{11}H_9N_2O_5$, 249.0511; found 249.0510.

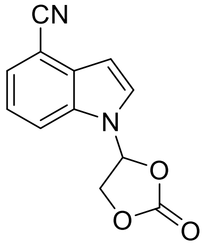

1-(2-oxo-1,3-dioxolan-4-yl)-1*H*-indole-4-carbonitrile (3m): Yield: 91%, 112.2 mg, white solid, mp 166-168 °C, R_f = 0.35 (H/E = 2:1). ^{1}H NMR (400 MHz, d_6-DMSO) δ 8.05 (d, J = 3.4 Hz, 1H), 7.99 (d, J = 8.4 Hz, 1H), 7.72 (d, J = 7.4 Hz, 1H), 7.46 (t, J = 7.9 Hz, 1H), 7.30 (t, J = 6.3 Hz, 1H), 6.85 (d, J = 3.3 Hz, 1H), 5.06 (d, J = 6.3 Hz, 2H). ^{13}C NMR (101 MHz, d_6-DMSO) δ 153.8, 135.9, 130.4, 129.6, 126.9, 123.4, 118.4, 116.0, 103.5, 102.8, 82.0, 68.6. HRMS (ESI) m/z: [M + H]$^+$ calcd for $C_{12}H_9N_2O_3$, 229.0613; found 229.0612.

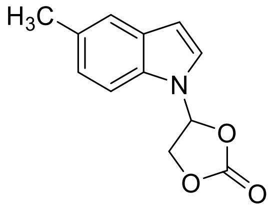

4-(5-methyl-1*H*-indol-1-yl)-1,3-dioxolan-2-one (3n): Yield: 51%, 55.6 mg, light yellow solid, mp 112-114 °C, R_f = 0.36 (H/E = 2:1). ^{1}H NMR (400 MHz, d_6-DMSO) δ 7.64 (d, J = 3.4 Hz, 1H), 7.47-7.40 (m, 2H), 7.18 (t, J = 6.3 Hz, 1H), 7.10 (d, J = 8.4 Hz, 1H), 6.58 (d, J = 3.3 Hz, 1H), 5.02 (d, J = 6.3 Hz, 2H), 2.39 (s, 3H). ^{13}C NMR (101 MHz, d_6-DMSO) δ 154.1, 134.3, 133.2, 130.3, 129.9, 126.3, 124.6, 121.2, 110.1, 105.1, 82.5, 68.3, 21.4. HRMS (ESI) m/z: [M + H]$^+$ calcd for $C_{12}H_{12}NO_3$, 218.0817; found 218.0810.

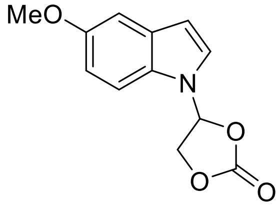

4-(5-methoxy-1*H*-indol-1-yl)-1,3-dioxolan-2-one (3o): Yield: 28%, 33.0 mg, white solid, mp 141-

143 °C, R_f = 0.32 (H/E = 2:1). ^{1}H NMR (400 MHz, $CDCl_3$) δ 7.32 (d, J = 8.6 Hz, 1H), 7.21 (d, J = 3.4 Hz, 1H), 7.15 (d, J = 1.9 Hz, 1H), 7.00 (dd, J = 8.9, 2.0 Hz, 1H), 6.73 (dd, J = 7.2, 5.1 Hz, 1H), 6.64 (d, J = 3.3 Hz, 1H), 4.98-4.85 (m, 2H), 3.91 (s, 3H). ^{13}C NMR (101 MHz, $CDCl_3$) δ 155.5, 153.3, 130.5, 130.2, 124.9, 113.4, 110.1, 106.2, 103.7, 82.2, 67.9, 55.8. HRMS (ESI) m/z: $[M + H]^+$ calcd for $C_{12}H_{12}NO_4$, 234.0766; found 234.0758.

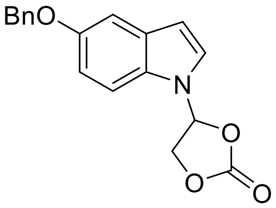

4-(5-(benzyloxy)-1*H*-indol-1-yl)-1,3-dioxolan-2-one (3p): Yield: 41%, 63.3 mg, white solid, mp 146-148 °C, R_f = 0.32 (H/E = 2:1). ^{1}H NMR (400 MHz, d_6-DMSO) δ 7.66 (d, J = 3.2 Hz, 1H), 7.47 (d, J = 8.1 Hz, 3H), 7.40 (t, J = 7.4 Hz, 2H), 7.33 (d, J = 6.9 Hz, 1H), 7.23 (s, 1H), 7.16 (t, J = 6.2 Hz, 1H), 7.00 (d, J = 8.9 Hz, 1H), 6.59 (d, J = 3.2 Hz, 1H), 5.13 (s, 2H), 5.01 (d, J = 6.3 Hz, 2H). ^{13}C NMR (101 MHz, d_6-DMSO) δ 154.10, 154.08, 137.9, 131.0, 130.2, 128.9, 128.2, 128.1, 126.9, 113.5, 111.1, 105.4, 105.0, 82.6, 70.1, 68.3. HRMS (ESI) m/z: $[M + H]^+$ calcd for $C_{18}H_{16}NO_4$, 310.1079; found 310.1078.

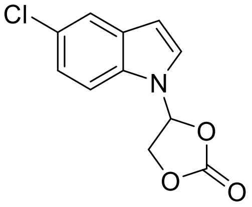

4-(5-chloro-1*H*-indol-1-yl)-1,3-dioxolan-2-one (3q): Yield: 85%, 100.4 mg, white solid, mp 127-129 °C, R_f = 0.37 (H/E = 2:1). ^{1}H NMR (400 MHz, d_6-DMSO) δ 7.81-7.79 (m, 1H), 7.70 (s, 1H), 7.62 (d, J = 8.8 Hz, 1H), 7.30 (d, J = 8.7 Hz, 1H), 7.22 (t, J = 6.3 Hz, 1H), 6.68 (d, J = 3.4 Hz, 1H), 5.04 (d, J = 6.3 Hz, 2H). ^{13}C NMR (101 MHz, d_6-DMSO) δ 154.0, 134.6, 130.7, 127.8, 126.1, 123.1, 120.8, 112.0, 105.2, 82.2, 68.5. HRMS (ESI) m/z: $[M + H]^+$ calcd for $C_{11}H_9ClNO_3$, 238.0271; found 238.0265.

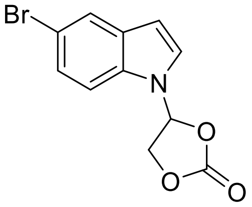

4-(5-bromo-1*H*-indol-1-yl)-1,3-dioxolan-2-one (3r): Yield: 80%, 112.5 mg, white solid, mp 108-110 °C, R_f = 0.37 (H/E = 2:1). ^{1}H NMR (400 MHz, d_6-DMSO) δ 7.85 (s, 1H), 7.79 (d, J = 3.3 Hz, 1H), 7.58 (d, J = 8.7 Hz, 1H), 7.42 (d, J = 8.6 Hz, 1H), 7.22 (t, J = 6.3 Hz, 1H), 6.68 (d, J = 3.2 Hz, 1H), 5.03 (d, J = 6.3 Hz, 2H). ^{13}C NMR (101 MHz, d_6-DMSO) δ 153.9, 134.9, 131.4, 127.7, 125.7, 123.8, 114.1, 112.5, 105.1, 82.2, 68.4. HRMS (ESI) m/z: $[M + H]^+$ calcd for $C_{11}H_9BrNO_3$, 281.9766; found 281.9757.

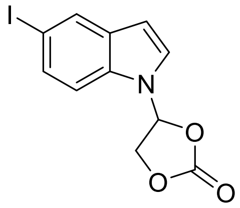

4-(5-iodo-1*H*-indol-1-yl)-1,3-dioxolan-2-one (3s): Yield: 86%, 141.6 mg, white solid, mp 134-136 °C, R_f = 0.36 (H/E = 2:1). ^{1}H NMR (400 MHz, d_6-DMSO) δ 8.02 (s, 1H), 7.73 (d, J = 3.3 Hz, 1H), 7.55 (d, J = 8.6 Hz, 1H), 7.45 (d, J = 8.7 Hz, 1H), 7.20 (t, J = 6.3 Hz, 1H), 6.65 (d, J = 3.2 Hz, 1H), 5.02 (d, J = 6.3 Hz, 2H). ^{13}C NMR (101 MHz, d_6-DMSO) δ 153.9, 135.3, 132.1, 131.1, 130.0, 127.2, 112.9, 104.8, 85.6, 82.1, 68.4. HRMS (ESI) m/z: [M + H]$^+$ calcd for $C_{11}H_9INO_3$, 329.9627; found 329.9619.

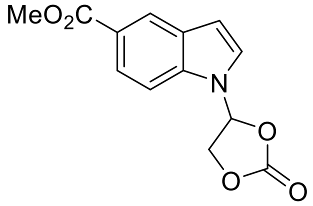

methyl 1-(2-oxo-1,3-dioxolan-4-yl)-1*H*-indole-5-carboxylate (3t): Yield: 82%, 107.2 mg, white solid, mp 164-166 °C, R_f = 0.34 (H/E = 2:1). ^{1}H NMR (400 MHz, d_6-DMSO) δ 8.33 (s, 1H), 7.90 (d, J = 8.7 Hz, 1H), 7.86 (d, J = 3.3 Hz, 1H), 7.71 (d, J = 8.7 Hz, 1H), 7.28 (t, J = 6.3 Hz, 1H), 6.85 (d, J = 3.3 Hz, 1H), 5.05 (d, J = 6.2 Hz, 2H), 3.87 (s, 3H). ^{13}C NMR (101 MHz, d_6-DMSO) δ 167.2, 153.9, 138.7, 129.2, 127.7, 124.0, 123.8, 123.1, 110.6, 106.7, 82.1, 68.6, 52.4. HRMS (ESI) m/z: [M + H]$^+$ calcd for $C_{13}H_{12}NO_5$, 262.0715; found 262.0708.

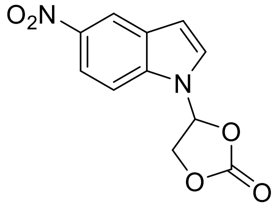

4-(5-nitro-1*H*-indol-1-yl)-1,3-dioxolan-2-one (3u): Yield: 91%, 112.5 mg, light yellow solid, mp 188-190 °C, R_f = 0.40 (H/E = 1:1). ^{1}H NMR (400 MHz, d_6-DMSO) δ 8.64 (s, 1H), 8.18 (d, J = 9.1 Hz, 1H), 8.00 (d, J = 3.4 Hz, 1H), 7.83 (d, J = 9.1 Hz, 1H), 7.32 (t, J = 6.3 Hz, 1H), 6.97 (d, J = 3.4 Hz, 1H), 5.12-4.99 (m, 2H). ^{13}C NMR (101 MHz, d_6-DMSO) δ 153.8, 142.6, 139.2, 129.5, 128.9, 118.4, 118.3, 111.2, 107.6, 81.9, 68.7. HRMS (ESI) m/z: [M + H]$^+$ calcd for $C_{11}H_9N_2O_5$, 249.0511; found 249.0503.

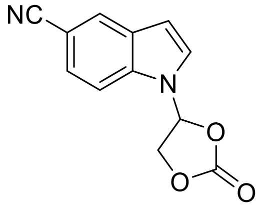

1-(2-oxo-1,3-dioxolan-4-yl)-1*H*-indole-5-carbonitrile (3v): Yield: 97%, 111.0 mg, white solid, mp 186-188 °C, R_f = 0.40 (H/E = 1:1). ^{1}H NMR (400 MHz, d_6-DMSO) δ 8.19 (s, 1H), 7.95 (d, J = 3.4 Hz, 1H), 7.81 (d, J = 8.6 Hz, 1H), 7.67 (d, J = 8.6 Hz, 1H), 7.30 (t, J = 6.2 Hz, 1H), 6.83 (d, J = 3.3 Hz, 1H), 5.05 (d, J = 5.8 Hz, 2H). ^{13}C NMR (101 MHz, d_6-DMSO) δ 153.8, 137.9, 129.3, 128.6, 127.0, 126.1,

120.5, 111.9, 106.2, 103.9, 81.9, 68.6. HRMS (ESI) m/z: $[M + H]^+$ calcd for $C_{12}H_9N_2O_3$, 229.0613; found 229.0607.

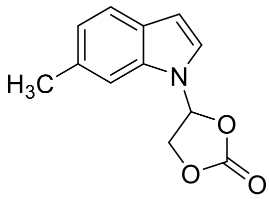

4-(6-methyl-1*H*-indol-1-yl)-1,3-dioxolan-2-one (3w): Yield: 47%, 51.2 mg, white solid, mp 130-132 °C, R_f = 0.36 (H/E = 2:1). ^{1}H NMR (400 MHz, d_6-DMSO) δ 7.61 (d, J = 3.4 Hz, 1H), 7.51 (d, J = 8.0 Hz, 1H), 7.38 (s, 1H), 7.18 (t, J = 6.3 Hz, 1H), 7.01 (d, J = 8.1 Hz, 1H), 6.61 (d, J = 3.2 Hz, 1H), 5.02 (d, J = 6.3 Hz, 2H), 2.44 (s, 3H). ^{13}C NMR (101 MHz, d_6-DMSO) δ 154.1, 133.2, 132.5, 127.3, 125.4, 123.2, 121.2, 110.3, 105.5, 82.3, 68.3, 22.0. $[M + H]^+$ calcd for $C_{12}H_{12}NO_3$, 218.0817; found 218.0810.

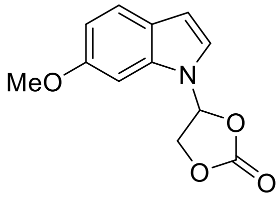

4-(6-methoxy-1*H*-indol-1-yl)-1,3-dioxolan-2-one (3x): Yield: 55%, 63.9 mg, white solid, mp 152-154 °C, R_f = 0.34 (H/E = 2:1). ^{1}H NMR (400 MHz, d_6-DMSO) δ 7.54 (d, J = 3.5 Hz, 1H), 7.49 (d, J = 8.6 Hz, 1H), 7.23 (t, J = 6.3 Hz, 1H), 7.19 (s, 1H), 6.82 (d, J = 8.6 Hz, 1H), 6.60 (d, J = 3.3 Hz, 1H), 5.02 (d, J = 6.3 Hz, 2H), 3.81 (s, 3H). ^{13}C NMR (101 MHz, d_6-DMSO) δ 157.0, 154.1, 137.2, 124.4, 123.2, 122.0, 111.2, 105.7, 94.4, 82.1, 68.4, 55.9. HRMS (ESI) m/z: $[M + H]^+$ calcd for $C_{12}H_{12}NO_4$, 234.0766; found 234.0765.

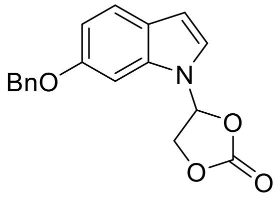

4-(6-(benzyloxy)-1*H*-indol-1-yl)-1,3-dioxolan-2-one (3y): Yield: 55%, 85.0 mg, white solid, mp 139-141 °C, R_f = 0.36 (H/E = 2:1). ^{1}H NMR (400 MHz, d_6-DMSO) δ 7.55 (d, J = 3.4 Hz, 1H), 7.52-7.48 (m, 3H), 7.41 (t, J = 7.4 Hz, 2H), 7.37-7.31 (m, 2H), 7.21 (t, J = 6.3 Hz, 1H), 6.90 (d, J = 8.7 Hz, 1H), 6.60 (d, J = 3.3 Hz, 1H), 5.19-5.12 (m, 2H), 5.02 (d, J = 6.3 Hz, 2H). ^{13}C NMR (101 MHz, d_6-DMSO) δ 156.0, 154.1, 137.6, 137.2, 133.2, 128.9, 128.3, 128.2, 124.6, 123.5, 122.1, 111.7, 105.7, 95.8, 82.1, 70.2, 68.3. HRMS (ESI) m/z: $[M + H]^+$ calcd for $C_{18}H_{16}NO_4$, 310.1079; found 310.1071.

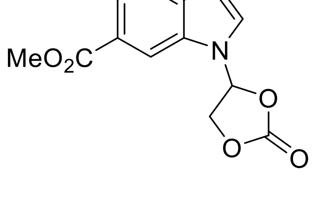

methyl 1-(2-oxo-1,3-dioxolan-4-yl)-1*H*-indole-6-carboxylate (3z): Yield: 90%, 116.9 mg, white solid, mp 140-142 °C, R_f = 0.37 (H/E = 2:1). ^{1}H NMR (400 MHz, d_6-DMSO) δ 8.28 (s, 1H), 7.98 (d, J = 3.3 Hz, 1H), 7.80-7.73 (m, 2H), 7.38 (t, J = 6.3 Hz, 1H), 6.80 (d, J = 3.3 Hz, 1H), 5.16-4.92 (m, 2H), 3.89

(s, 3H). ^{13}C NMR (101 MHz, d_6-DMSO) δ 167.3, 153.9, 135.6, 133.2, 129.7, 124.3, 122.2, 121.5, 112.2, 105.87, 82.0, 68.5, 52.5. HRMS (ESI) m/z: [M + H]$^+$ calcd for $C_{13}H_{12}NO_5$, 262.0715; found 262.0712.

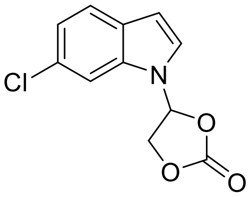

4-(6-chloro-1*H*-indol-1-yl)-1,3-dioxolan-2-one (3aa): Yield: 92%, 109.0 mg, white solid, mp 152-154 °C, R_f = 0.38 (H/E = 2:1). ^{1}H NMR (400 MHz, d_6-DMSO) δ 7.76 (s, 2H), 7.64 (d, J = 8.4 Hz, 1H), 7.30-7.17 (m, 2H), 6.72 (d, J = 3.3 Hz, 1H), 5.03 (d, J = 6.0 Hz, 2H). ^{13}C NMR (101 MHz, d_6-DMSO) δ 153.9, 136.7, 133.2, 128.1, 128.0, 126.9, 122.9, 121.9, 110.6, 105.9, 82.0, 68.5. HRMS (ESI) m/z: [M + H]$^+$ calcd for $C_{11}H_9ClNO_3$, 238.0271; found 238.0264.

3. Substrate Extension Studies.

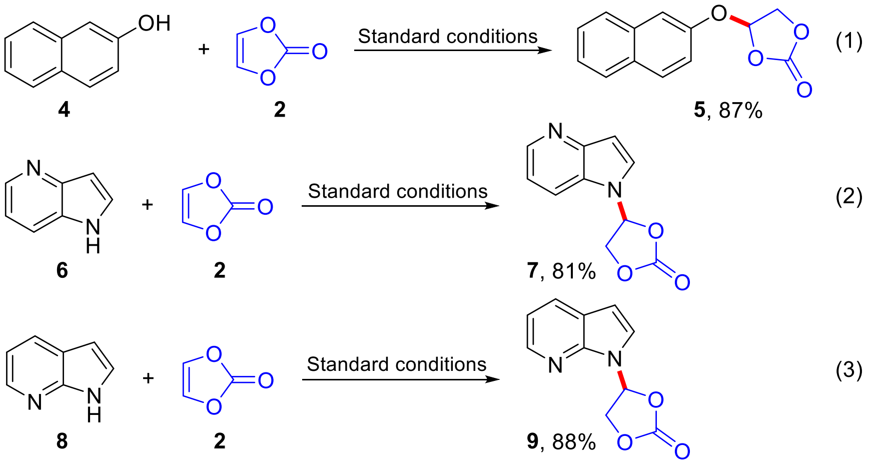

Method A: To an oven-dried 25 mL Schlenk tube equipped with a magnetic stir bar was added 2-naphthol substrate **4** (0.50 mmol, 1.0 equiv.), vinylene carbonate (172 mg, 2.0 mmol, 4.0 equiv.) and K_2CO_3 (27.6 mg, 0.20 mmol, 40 mol%), and CH_3CN (3 mL) under an air atmosphere. The reaction mixture was stirred at 60 °C for 24 h, and then cooled to room temperature. The solvent was removed under reduced pressure and the crude product was purified by silica gel column chromatography to afford the desired products **5** as a white solid (100.0 mg, yield: 87%).

Method B: To an oven-dried 25 mL Schlenk tube equipped with a magnetic stir bar was added pyrrolo-pyridine substrate **6** or **8** (0.50 mmol, 1.0 equiv.), vinylene carbonate (172 mg, 2.0 mmol, 4.0 equiv.) and K_2CO_3 (27.6 mg, 0.20 mmol, 40 mol%), and CH_3CN (3 mL) under an air atmosphere. The reaction mixture was stirred at 60 °C for 24 h, and then cooled to room temperature. The solvent was removed under reduced pressure and the crude product was purified by silica gel column chromatography to afford the desired products **7** or **9**.

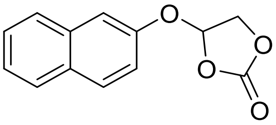

4-(naphthalen-2-yloxy)-1,3-dioxolan-2-one (5): Yield: 87%, 100.0 mg, white solid, mp 106-108 °C, R_f = 0.45 (H/E = 2:1). ^{1}H NMR (400 MHz, d_6-DMSO) δ 7.96 (d, J = 8.9 Hz, 1H), 7.92 (d, J = 8.2 Hz, 2H), 7.59 (s, 1H), 7.54 (t, J = 7.5 Hz, 1H), 7.46 (t, J = 7.5 Hz, 1H), 7.32 (dd, J = 8.9, 2.3 Hz, 1H), 6.71 (d, J = 3.9 Hz, 1H), 4.88 (dd, J = 9.9, 5.5 Hz, 1H), 4.66 (d, J = 10.0 Hz, 1H). ^{13}C NMR (101 MHz, d_6-DMSO) δ 154.1, 153.3, 134.1, 130.5, 130.2, 128.1, 127.7, 127.3, 125.4, 118.9, 111.3, 98.1, 70.9. HRMS (ESI) m/z: $[M + H]^+$ calcd for $C_{13}H_{11}O_4$, 231.0657; found 231.0654.

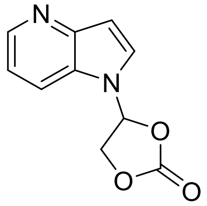

4-(1*H*-pyrrolo[3,2-b]pyridin-1-yl)-1,3-dioxolan-2-one (7): Yield: 81%, 82.2 mg, white solid, mp 176-178 °C, R_f = 0.31 (EtOAc). ^{1}H NMR (400 MHz, d_6-DMSO) δ 8.47 (d, J = 4.7 Hz, 1H), 8.05 (d, J = 3.5 Hz, 1H), 8.00 (d, J = 8.3 Hz, 1H), 7.29 (dd, J = 8.3, 4.6 Hz, 1H), 7.23 (t, J = 6.3 Hz, 1H), 6.81 (d, J = 3.4 Hz, 1H), 5.05 (d, J = 6.2 Hz, 2H). ^{13}C NMR (101 MHz, d_6-DMSO) δ 153.9, 147.6, 144.8, 129.8, 128.9, 118.0, 117.9, 106.1, 82.3, 68.4. HRMS (ESI) m/z: $[M + H]^+$ calcd for $C_{10}H_9N_2O_3$, 205.0613; found 205.0613.

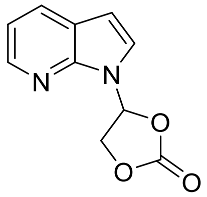

4-(1*H*-pyrrolo[2,3-b]pyridin-1-yl)-1,3-dioxolan-2-one (9): Yield: 88%, 89.3 mg, white solid, mp 169-171 °C, R_f = 0.37 (H/E = 2:1). ^{1}H NMR (400 MHz, d_6-DMSO) δ 8.33 (d, J = 4.7 Hz, 1H), 8.07 (d, J = 7.8 Hz, 1H), 7.82 (d, J = 3.7 Hz, 1H), 7.28-7.12 (m, 2H), 6.66 (d, J = 3.7 Hz, 1H), 5.08-4.98 (m, 2H). ^{13}C NMR (101 MHz, d_6-DMSO) δ 154.3, 147.5, 143.8, 130.0, 128.0, 121.9, 118.0, 102.9, 81.6, 68.4. HRMS (ESI) m/z: $[M + H]^+$ calcd for $C_{10}H_9N_2O_3$, 205.0613; found 205.0608.

4. X-Ray Crystallographic Analysis.

The structure of **3a** was determined based on single-crystal X-ray analysis. The detail procedure was shown as following: The **3a** solid was dissolved in AcOEt (1 mL). Then, the solvent was placed in the inner tube and *n*-hexane (5 mL) in the outer container. The crystals of **3a** were grown from solution, which is suitable for X-ray diffraction analysis.

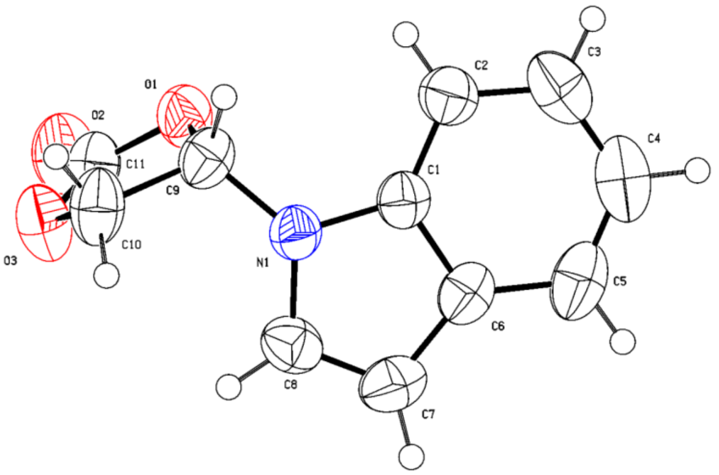

Figure S1. X-ray structure of **3a**.

CCDC No. 2299714 (**3a**) contains the supplementary crystallographic data for this paper. The crystal data can be obtained free of charge from the Cambridge Crystallographic Data Centre through www.ccdc.cam.ac.uk/data request/cif.

Table S1. Crystal data and structure refinement for **3a**.

CCDC number	2299714
Empirical formula	$C_{11}H_9NO_3$
Formula weight	203.19
Temperature	296(2) K
Wavelength (Å)	0.71073
Crystal system	Monoclinic
Space group	$P2_1/c$
a, b, c (Å)	10.247(3), 10.494(3), 9.560(3)
α, β, γ (°)	90, 112.873(6), 90
Volume (Å^3)	947.2(4)
Z	4
Density (calculated) (g/cm^3)	1.425
Absorption coefficient (mm^{-1})	0.105
F(000)	424
Crystal size (mm^3)	0.200×0.200×0.200
Theta range for data collection (°)	2.902 to 25.078
Index ranges	-12<=h<=12, -11<=k<=12, -11<=l<=11

Reflections collected	17596
Independent reflections	1673 [R(int) = 0.0717]
Completeness to theta = 25.057°	99.5 %
Absorption correction	Semi-empirical from equivalents
Refinement method	Full-matrix least-squares on F^2
Data / restraints / parameters	1673 / 0 / 136
Goodness-of-fit on F^2	1.061
Final R indices [$I>2\delta(I)$]	R1 = 0.0467, wR2 = 0.1222
R indices (all data)	R1 = 0.0708, wR2 = 0.1414
δLargest diff. peak and hole (e.Å^{-3})	0.134 and -0.177

5. Copy of NMR for the Products

^{1}H NMR of 3a

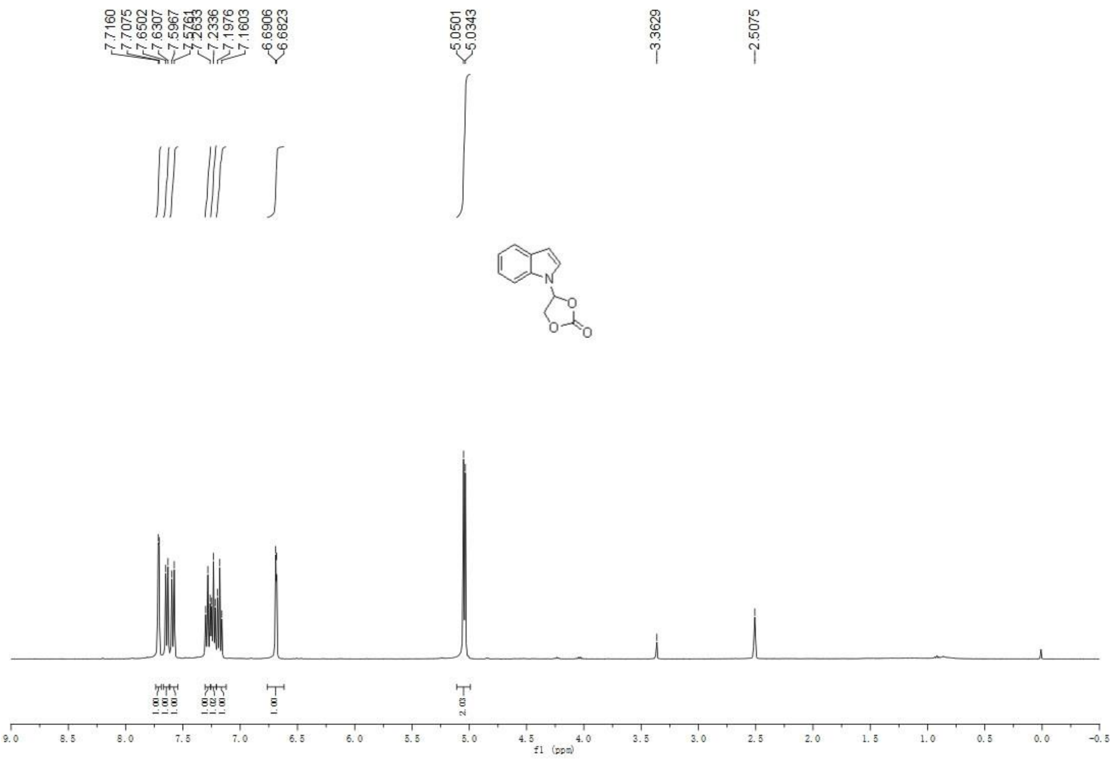

^{13}C NMR of 3a

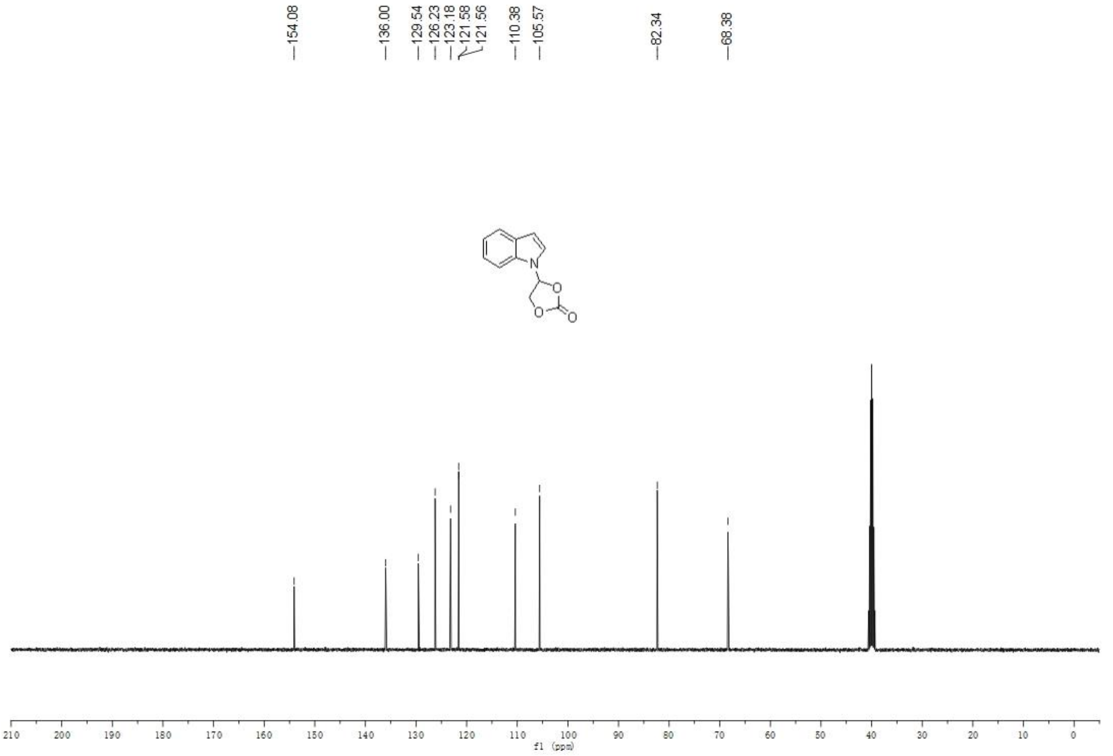

^{1}H NMR of 3e

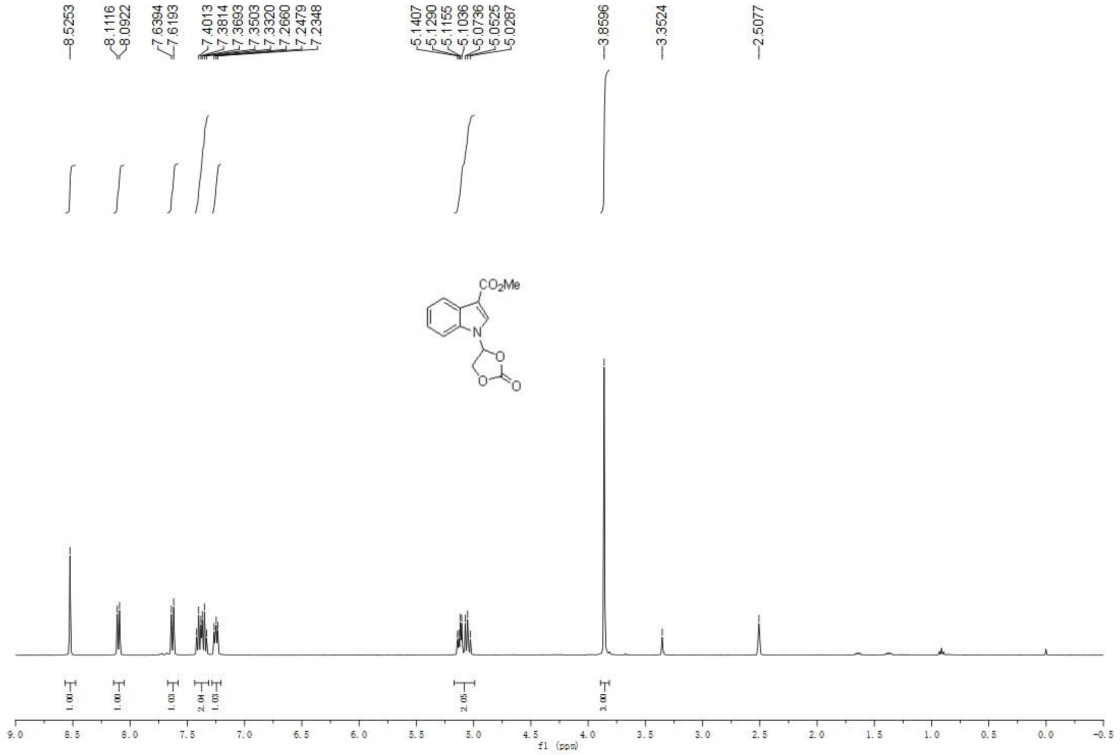

^{13}C NMR of 3e

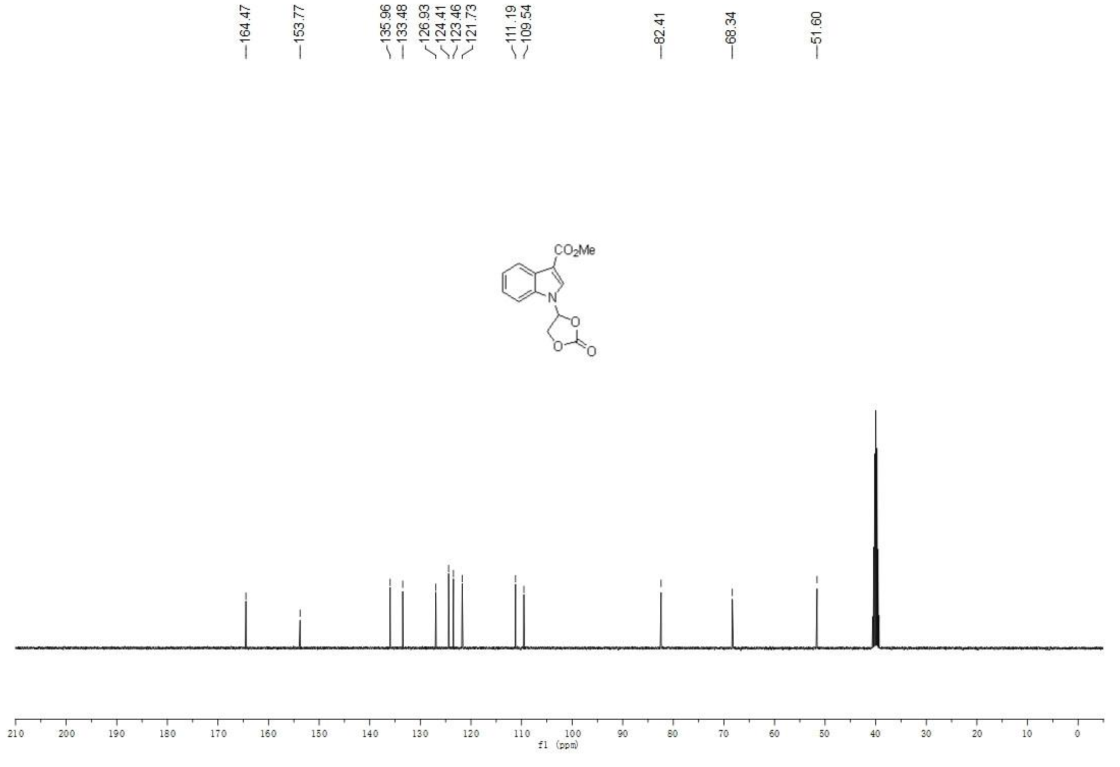

^{1}H NMR of 3f

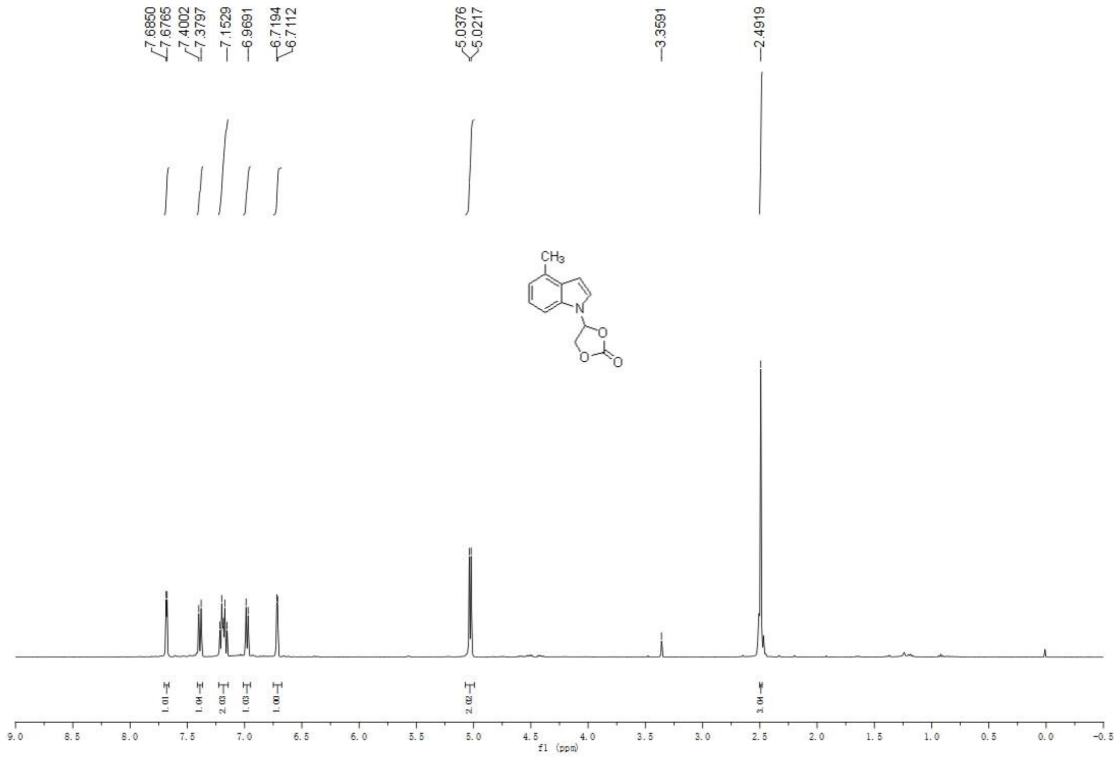

^{13}C NMR of 3f

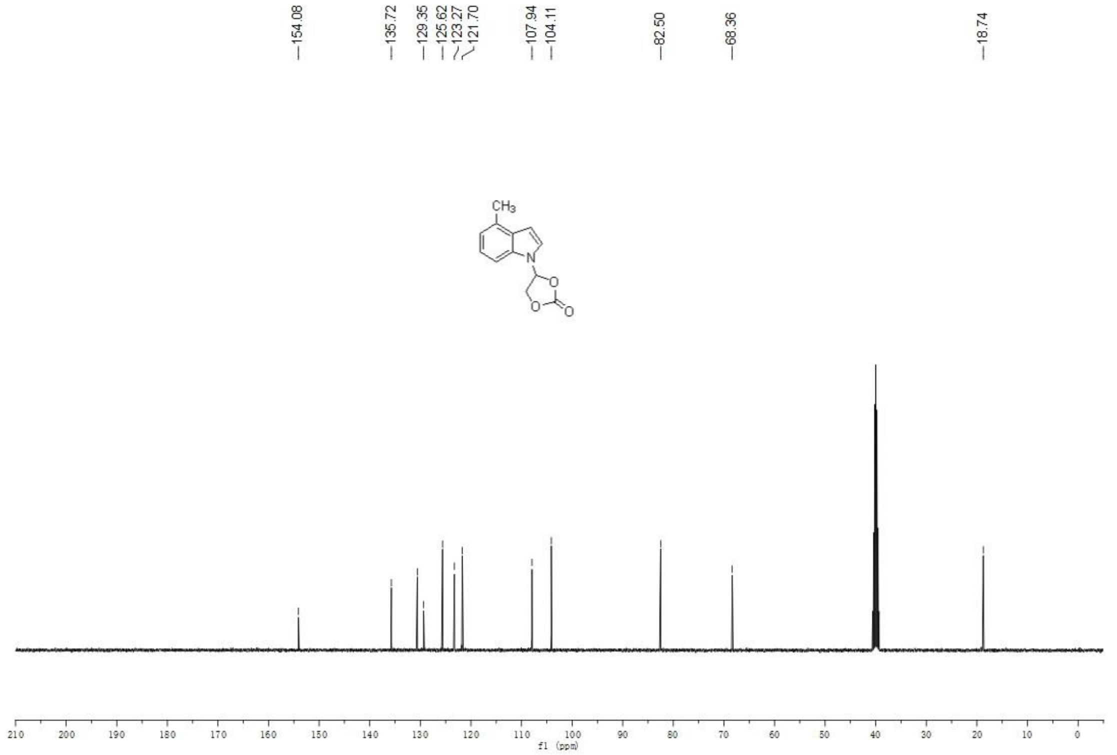

^{1}H NMR of 3g

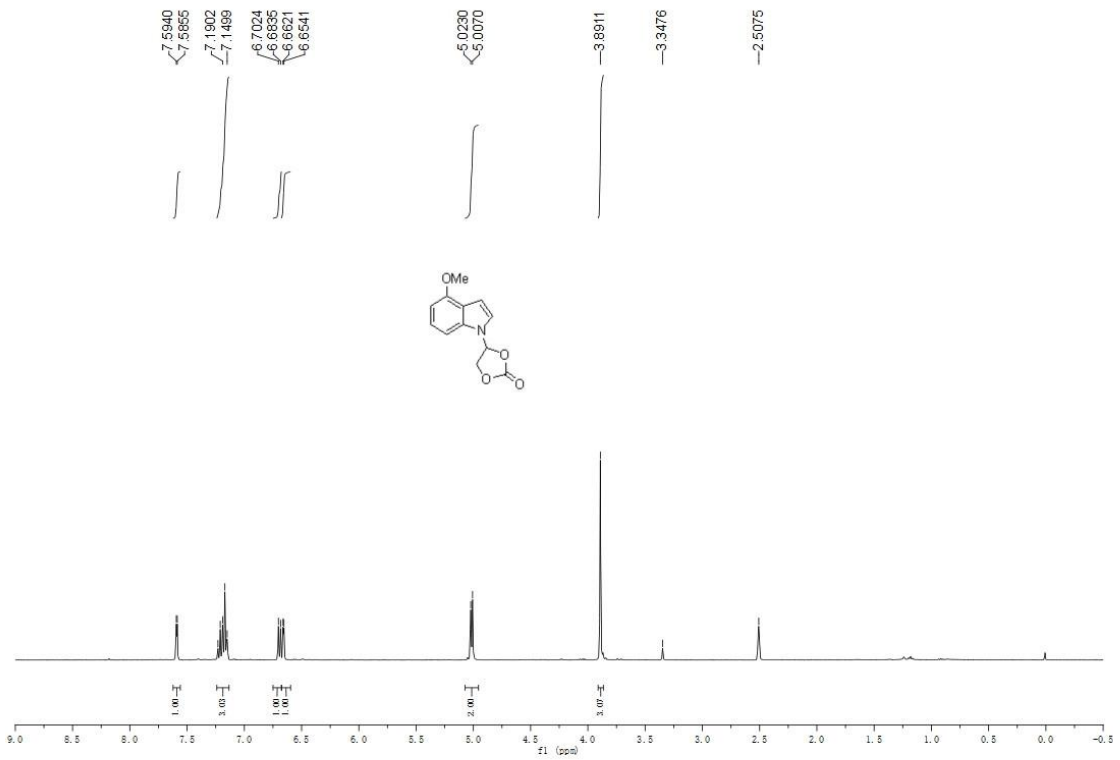

^{13}C NMR of 3g

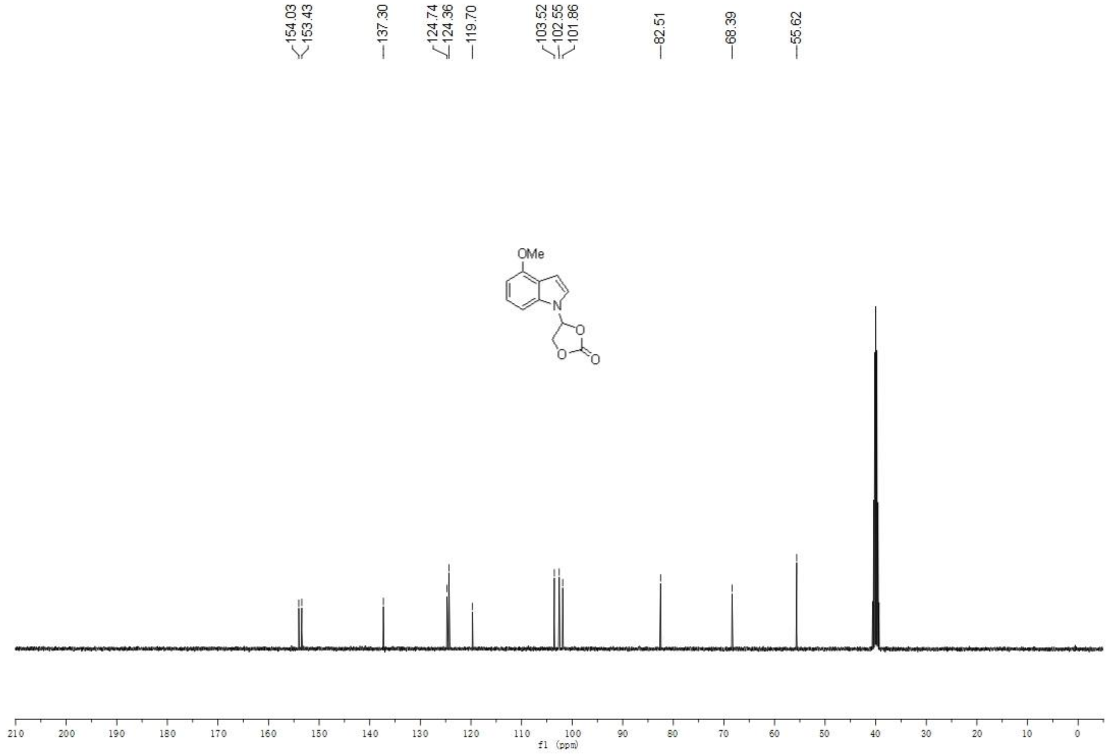

^{1}H NMR of 3h

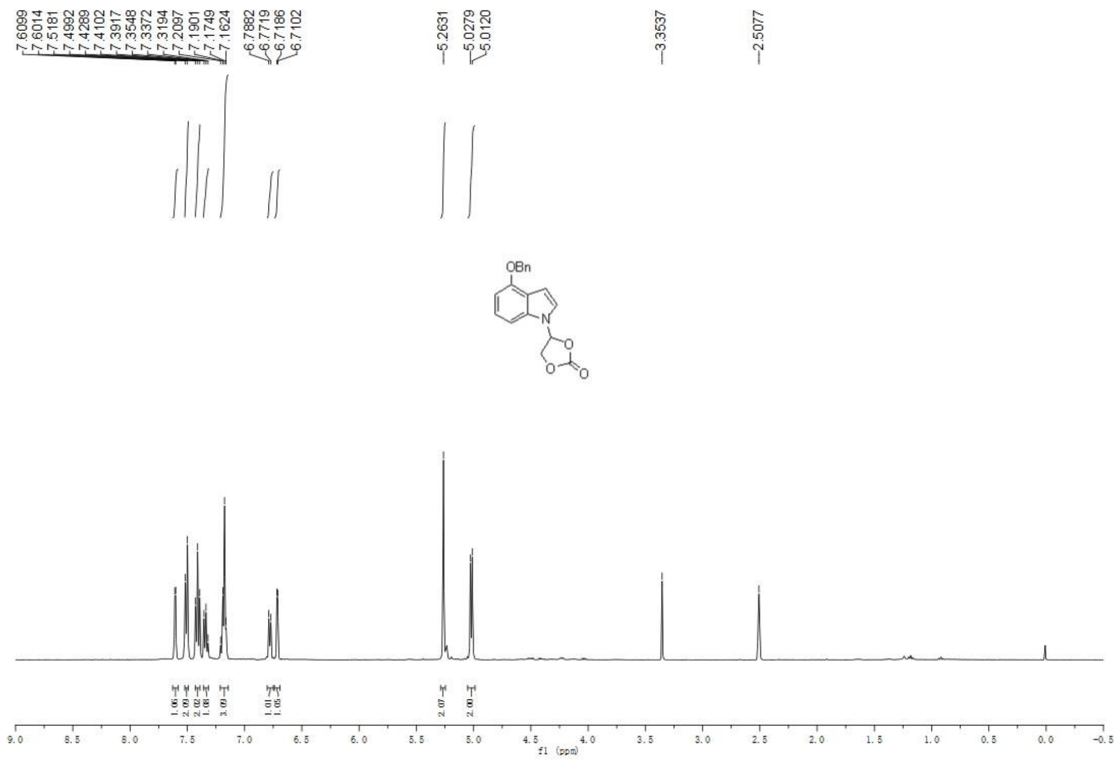

^{13}C NMR of 3h

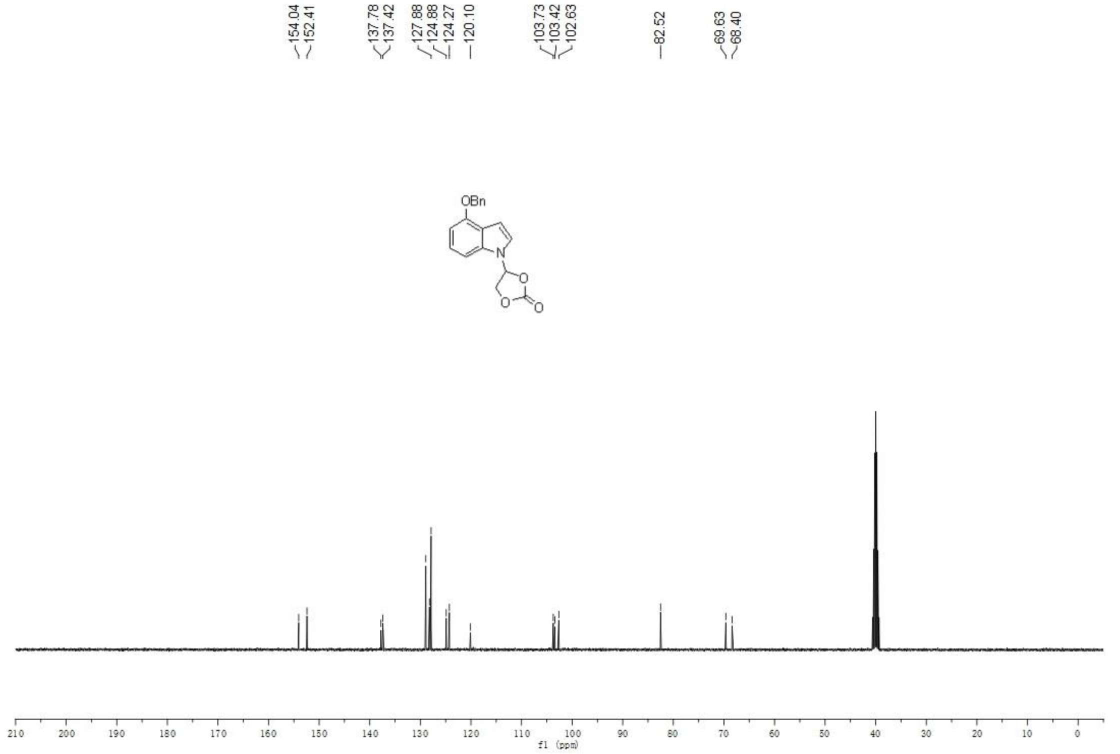

^{1}H NMR of 3i

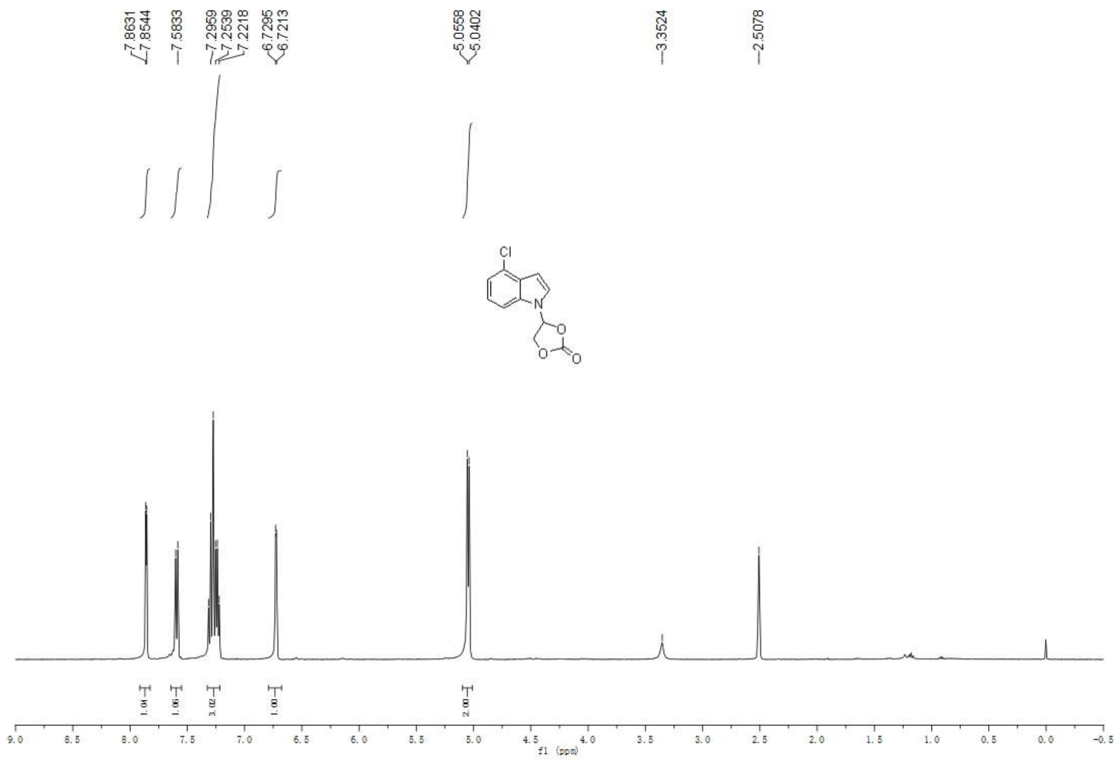

^{13}C NMR of 3i

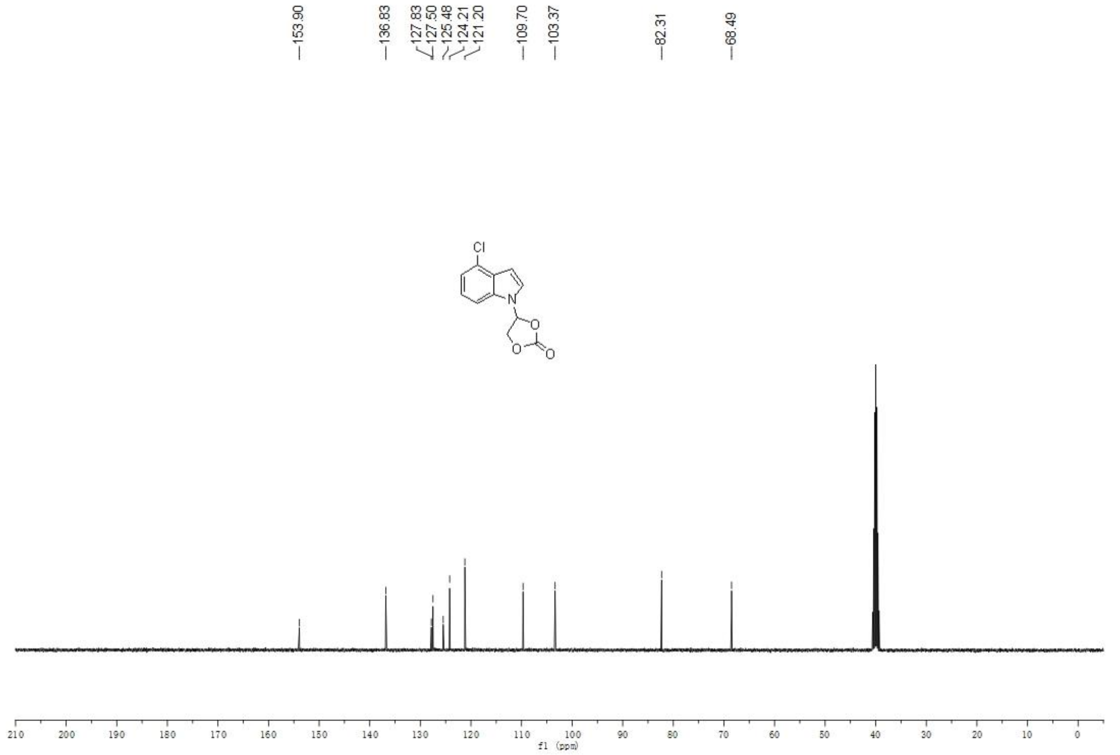

^{1}H NMR of 3j

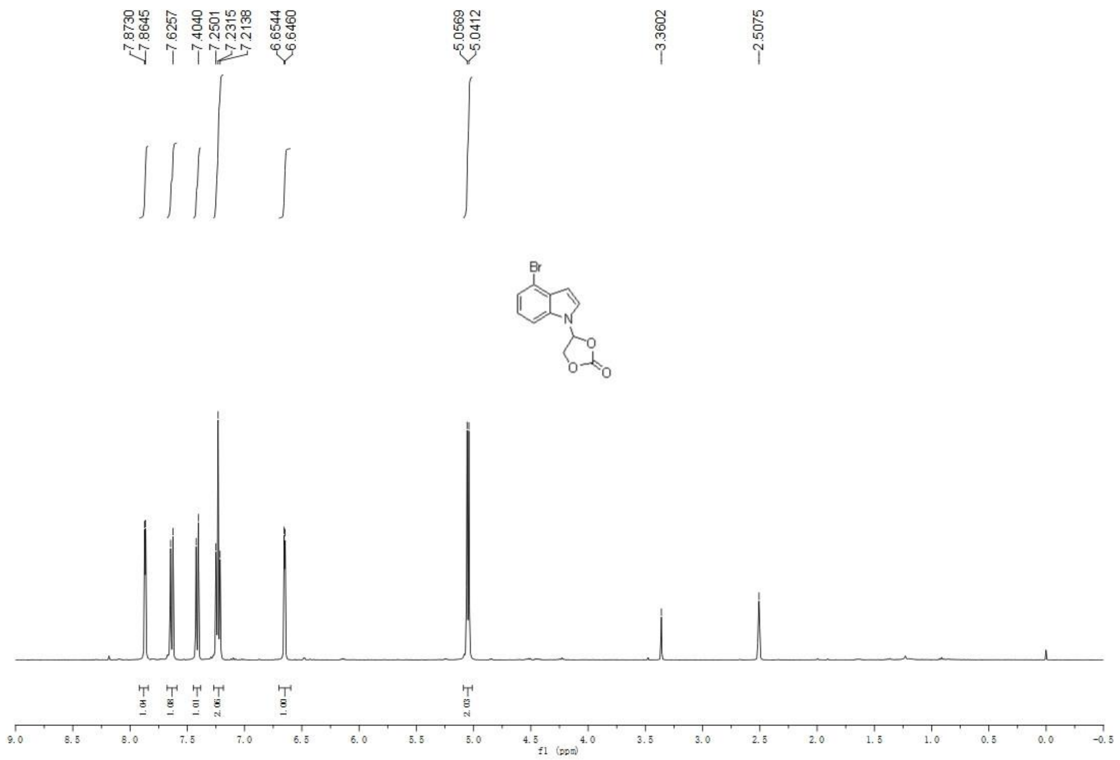

^{13}C NMR of 3j

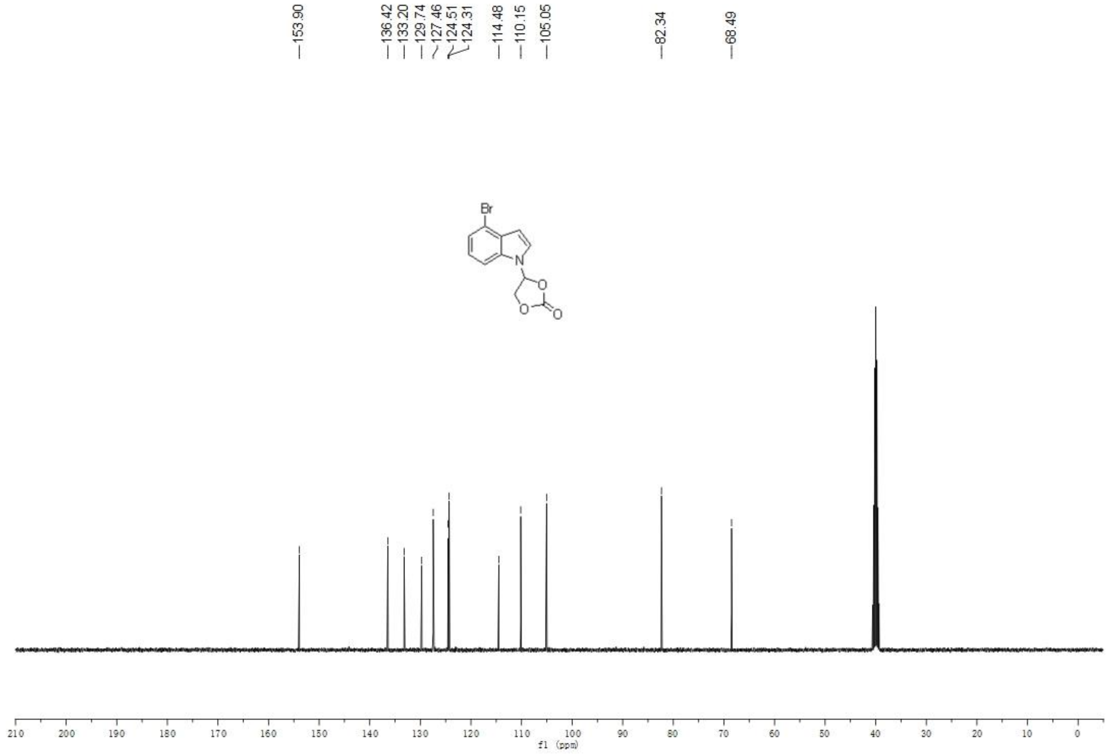

^{1}H NMR of 3k

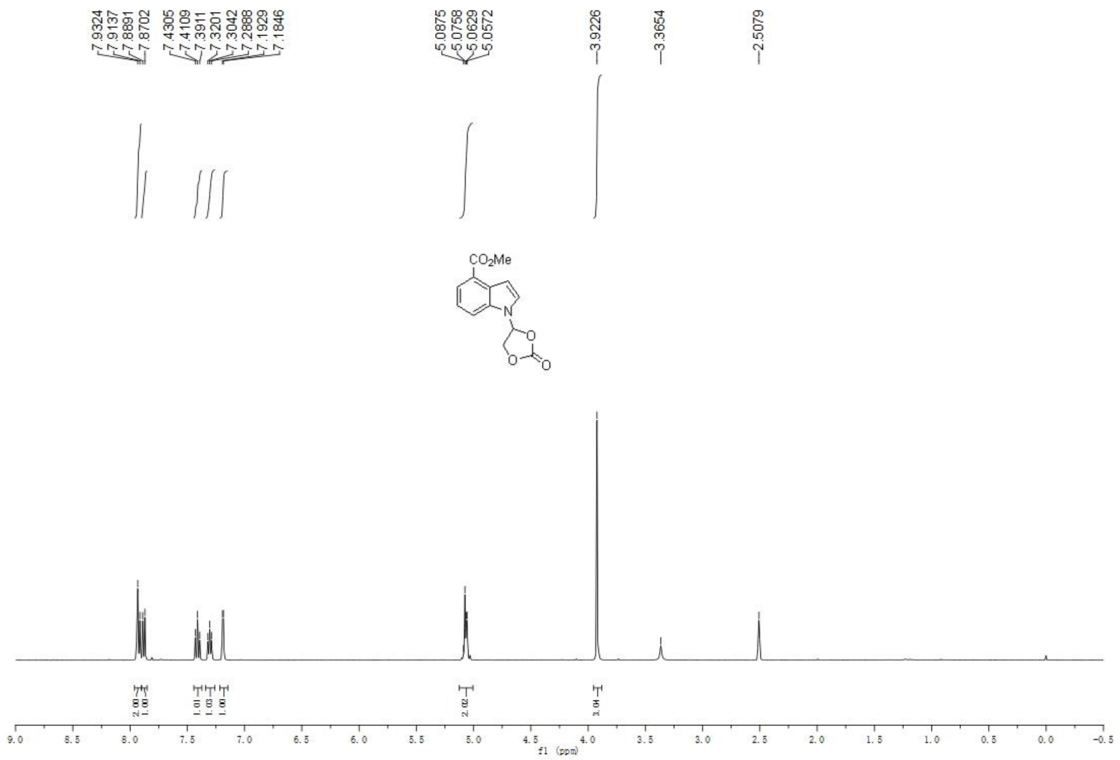

^{13}C NMR of 3k

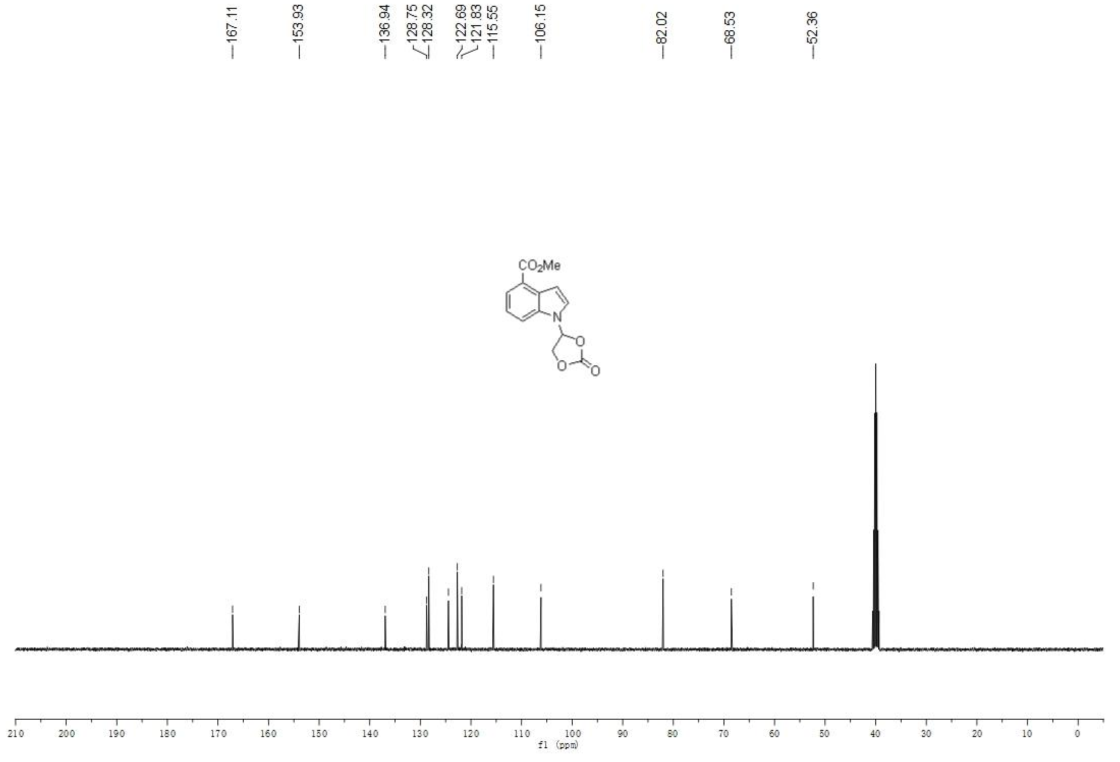

^{1}H NMR of 3l

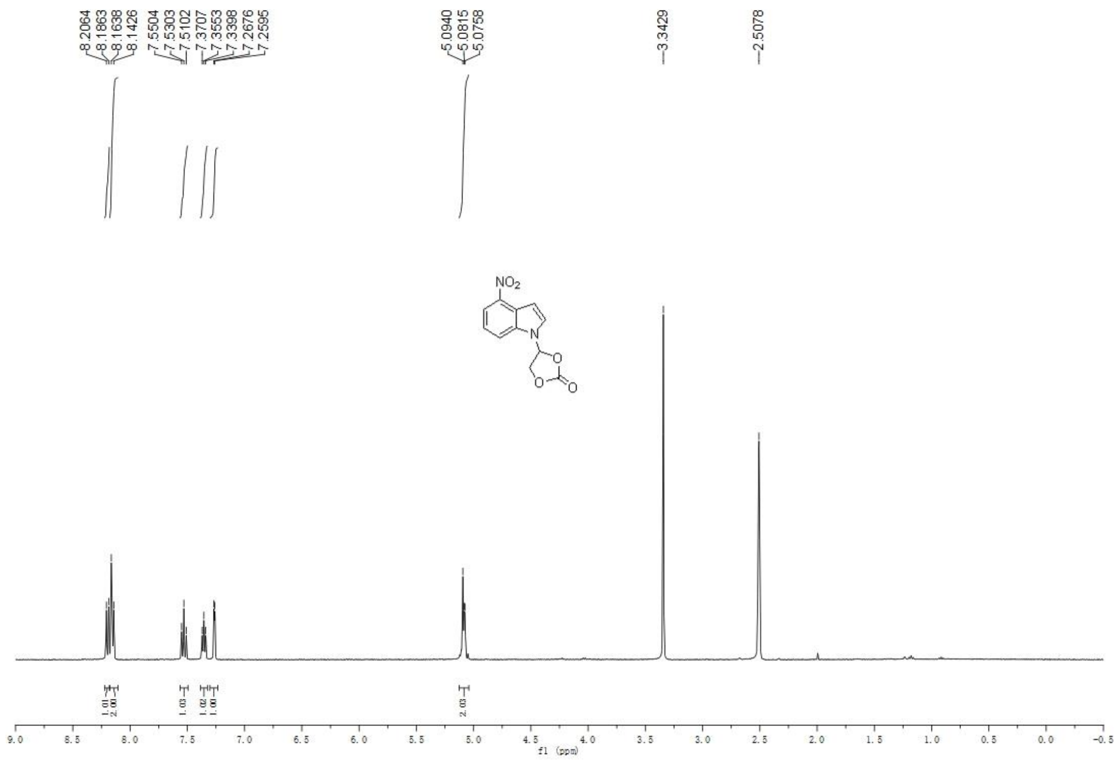

^{13}C NMR of 3l

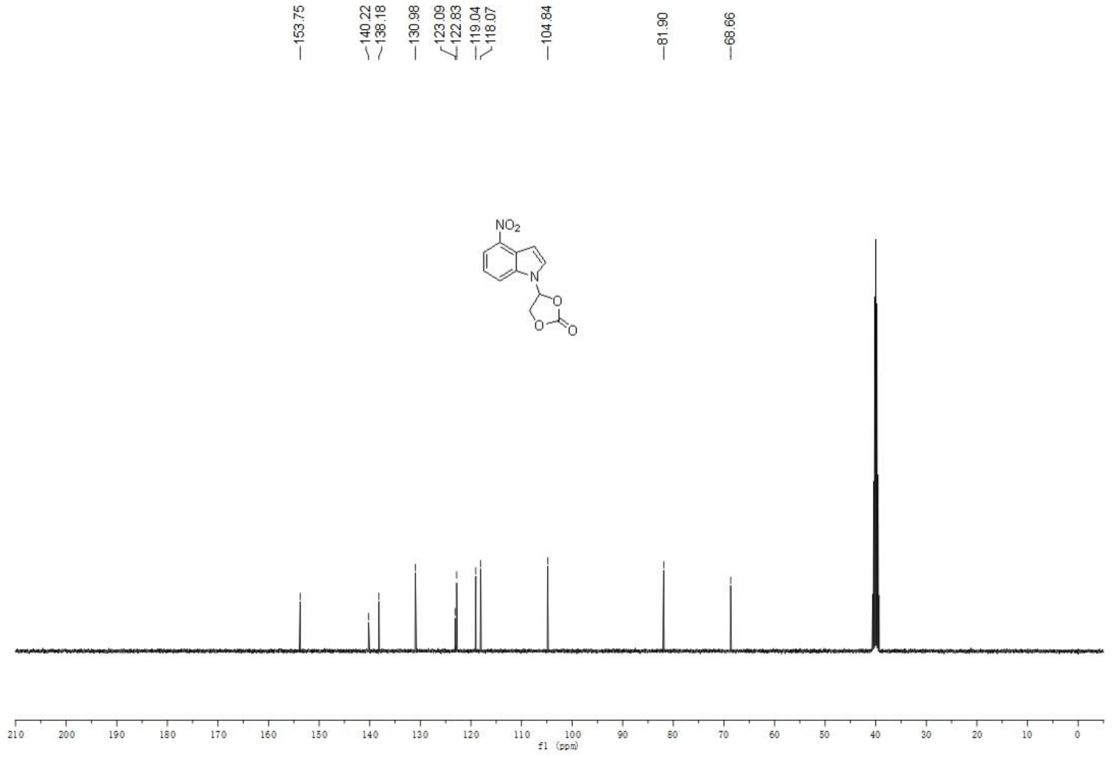

^{1}H NMR of 3m

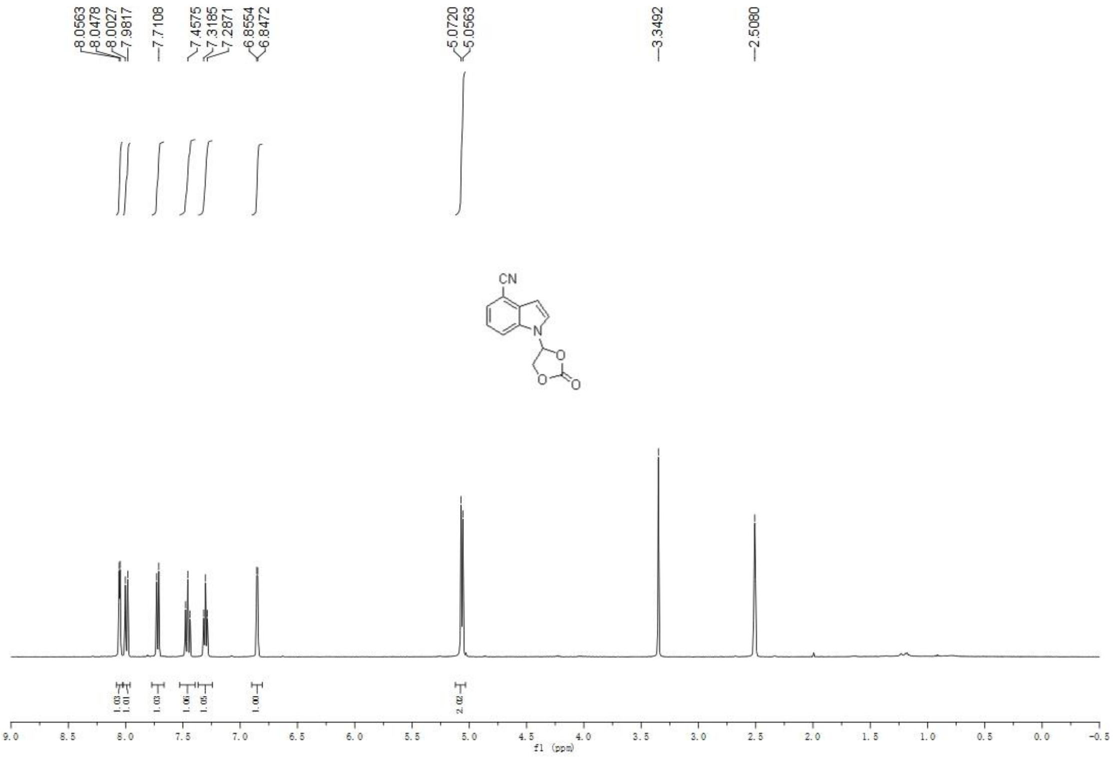

^{13}C NMR of 3m

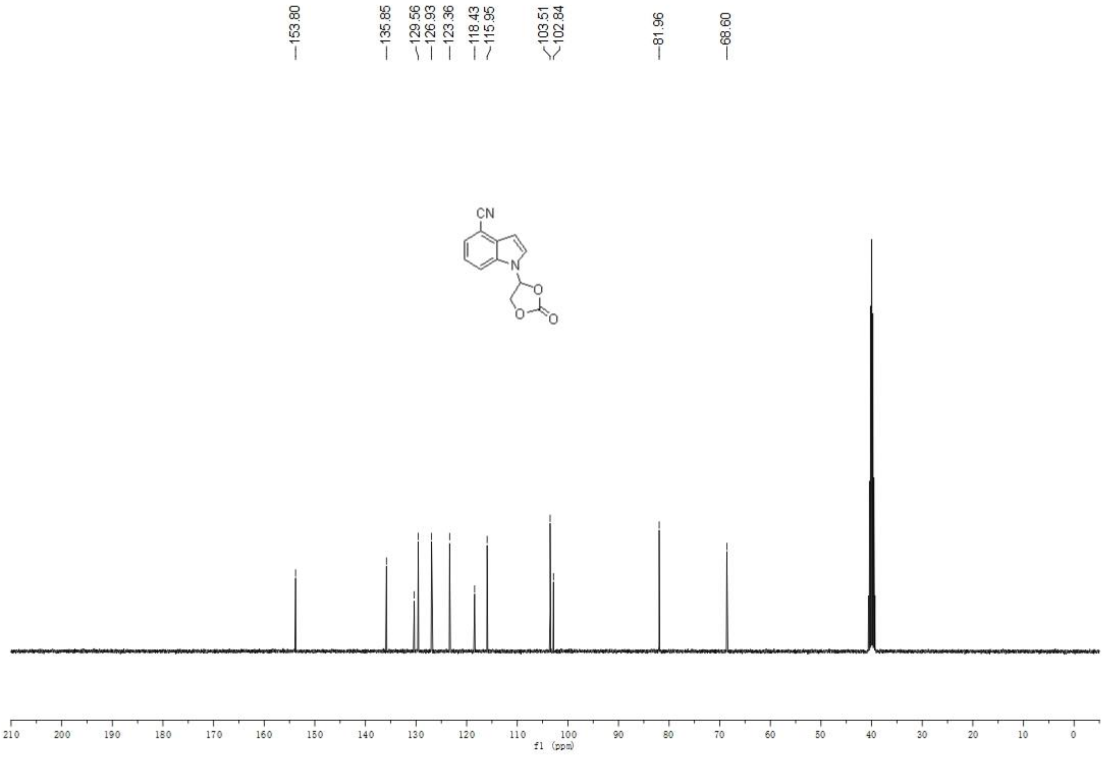

^{1}H NMR of 3n

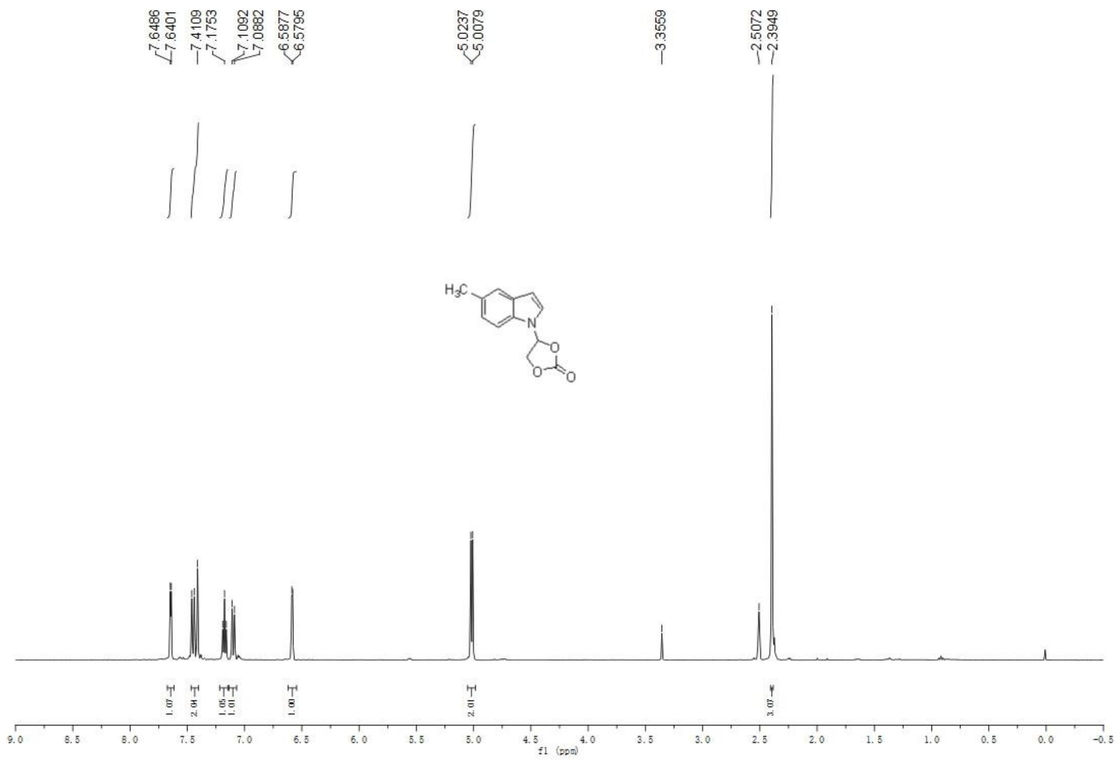

^{13}C NMR of 3n

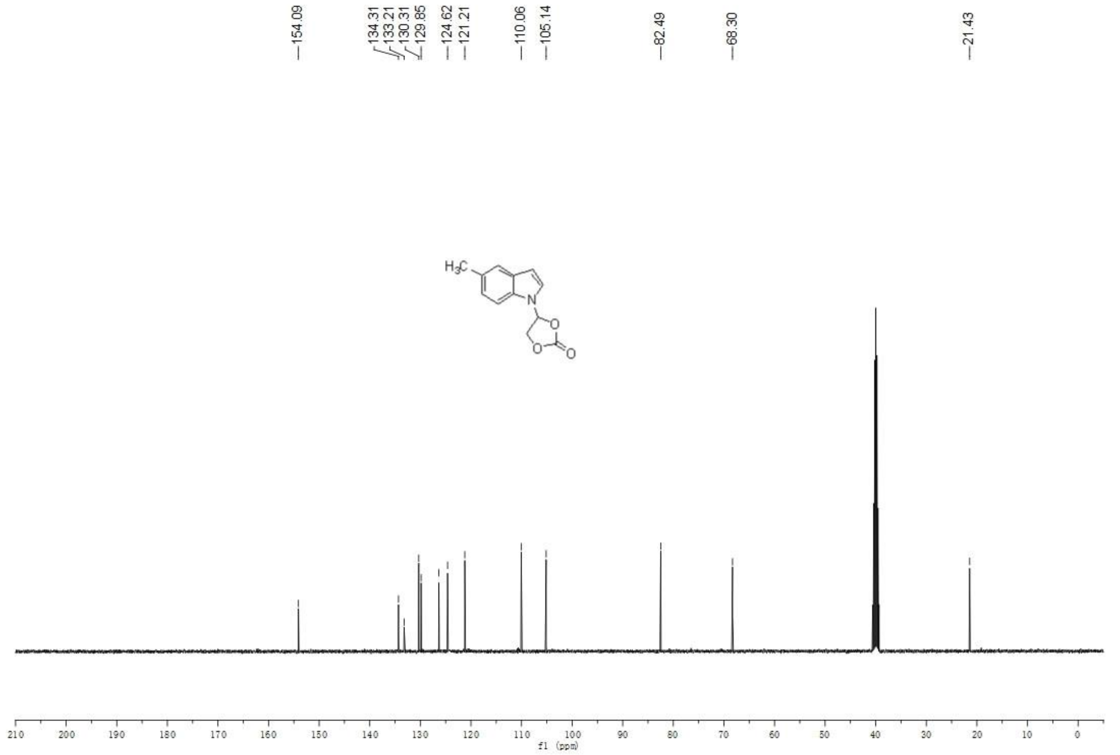

^{1}H NMR of 3o

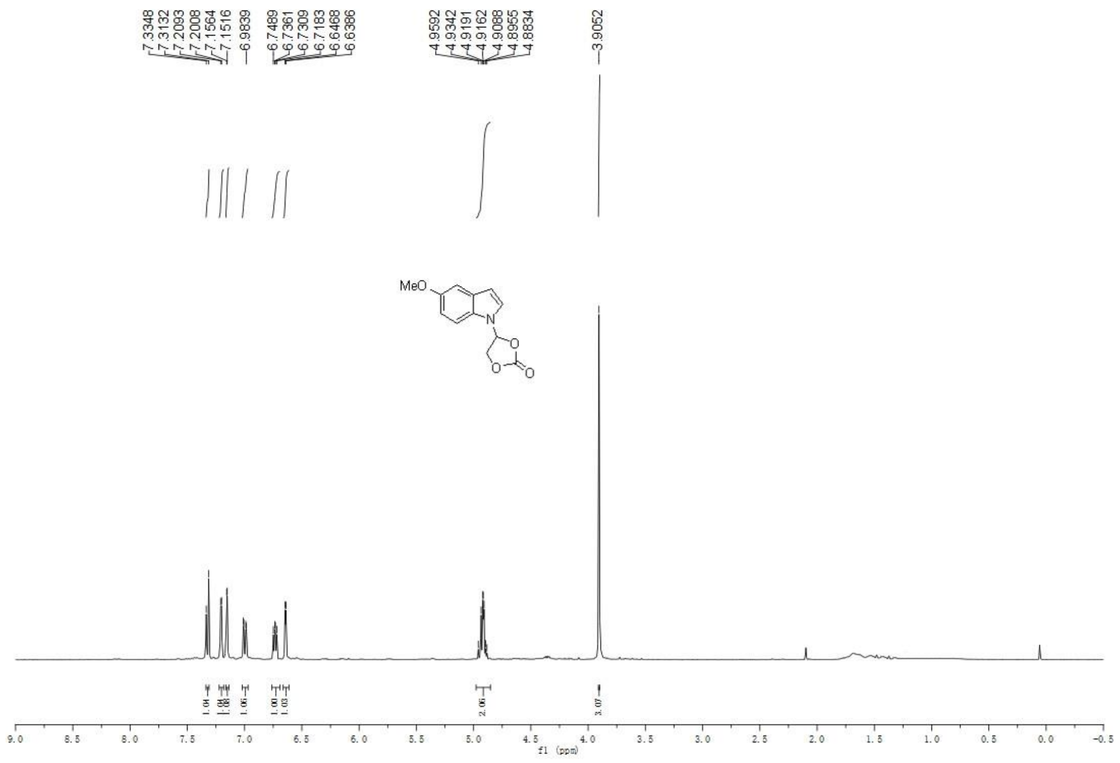

^{13}C NMR of 3o

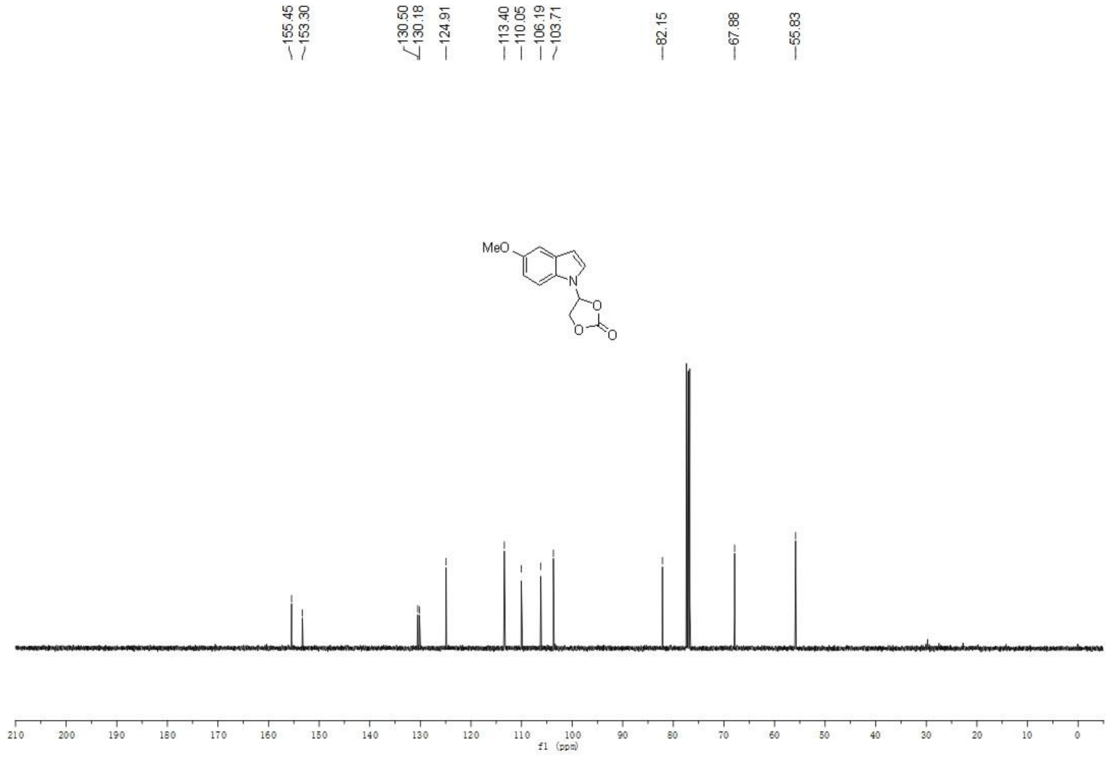

^{1}H NMR of 3p

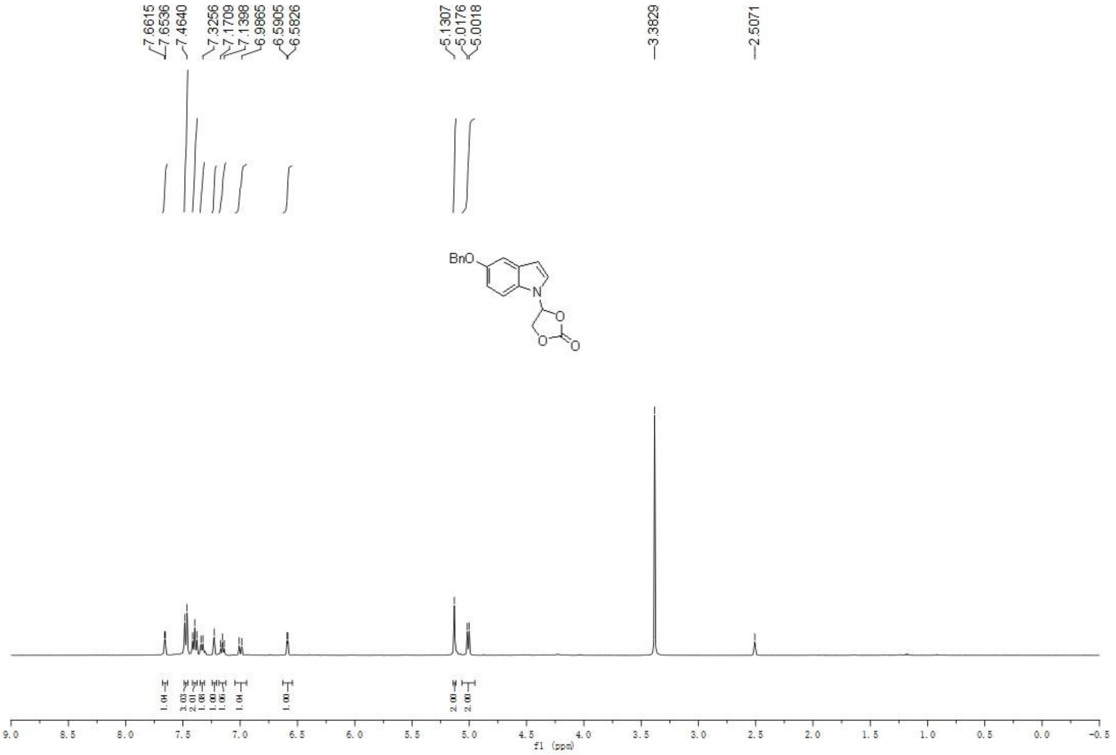

^{13}C NMR of 3p

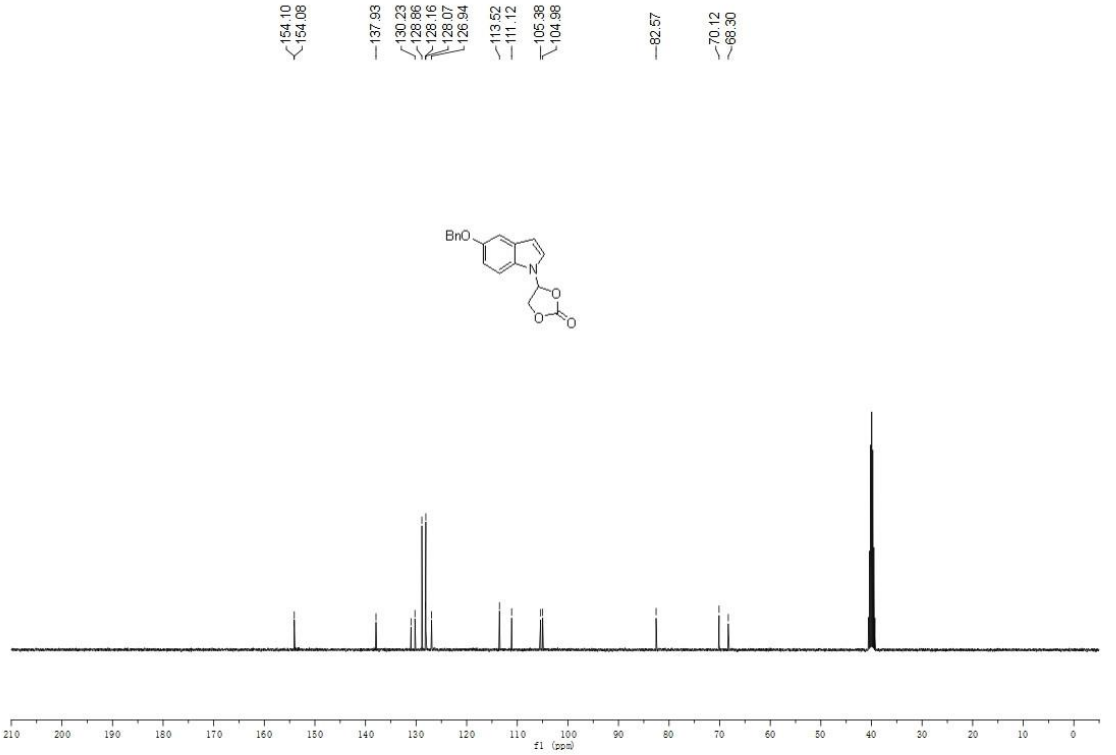

^{1}H NMR of 3q

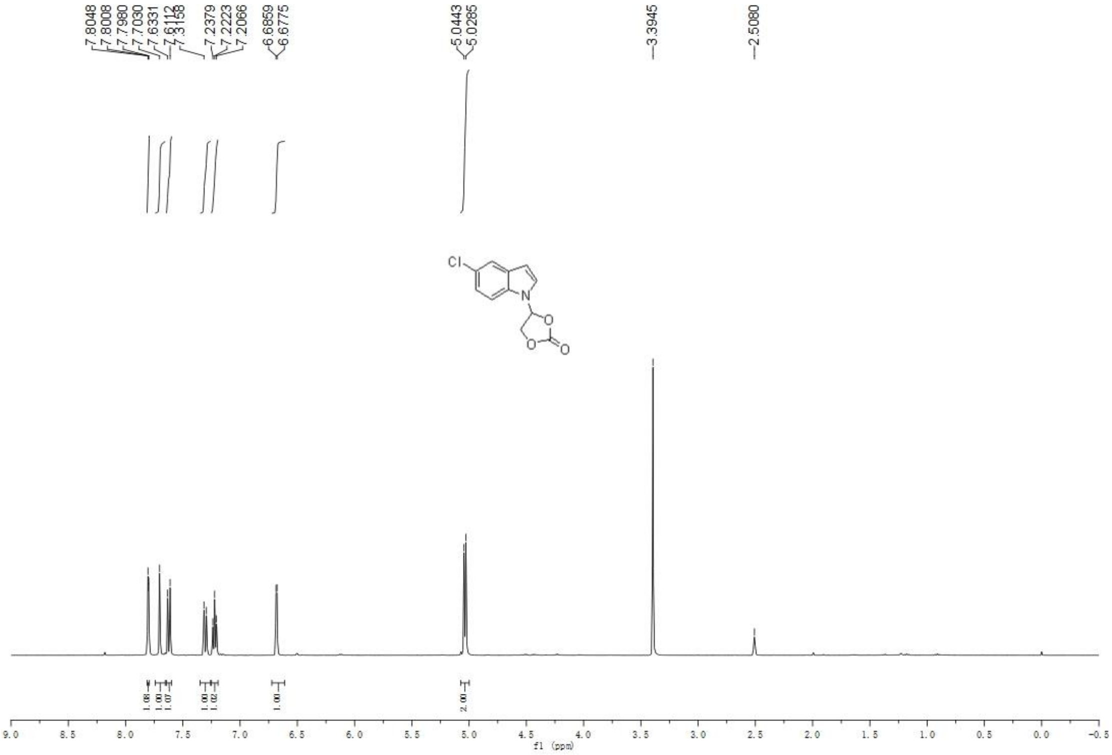

^{13}C NMR of 3q

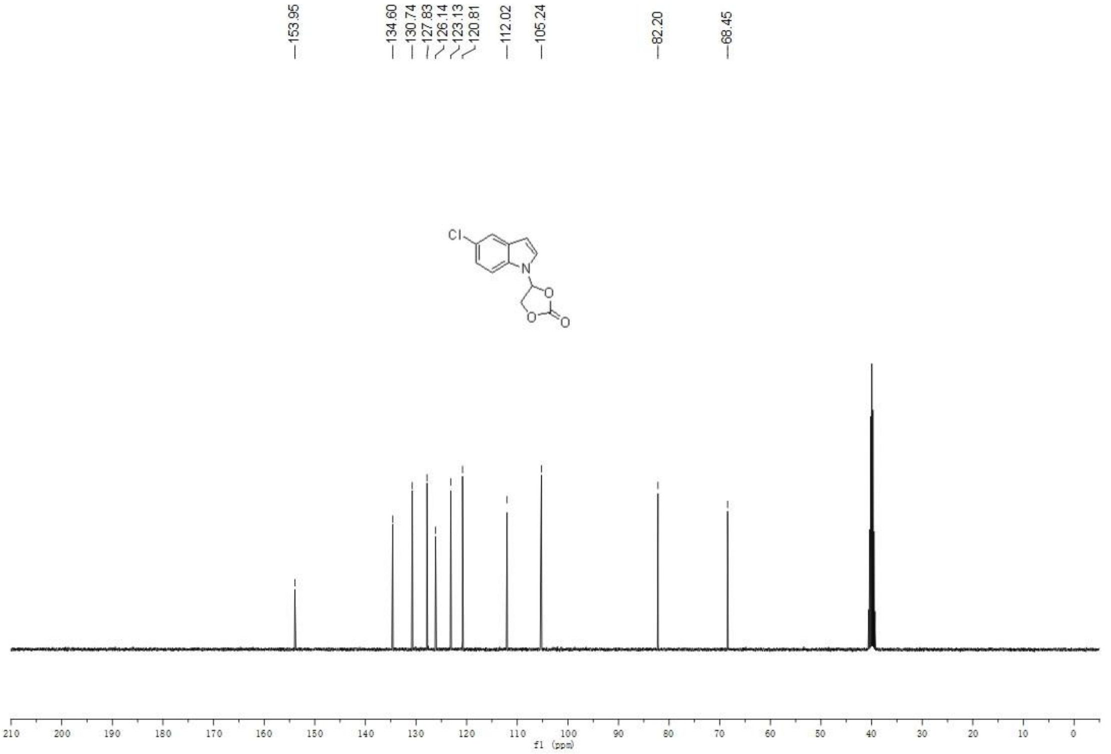

^{1}H NMR of 3r

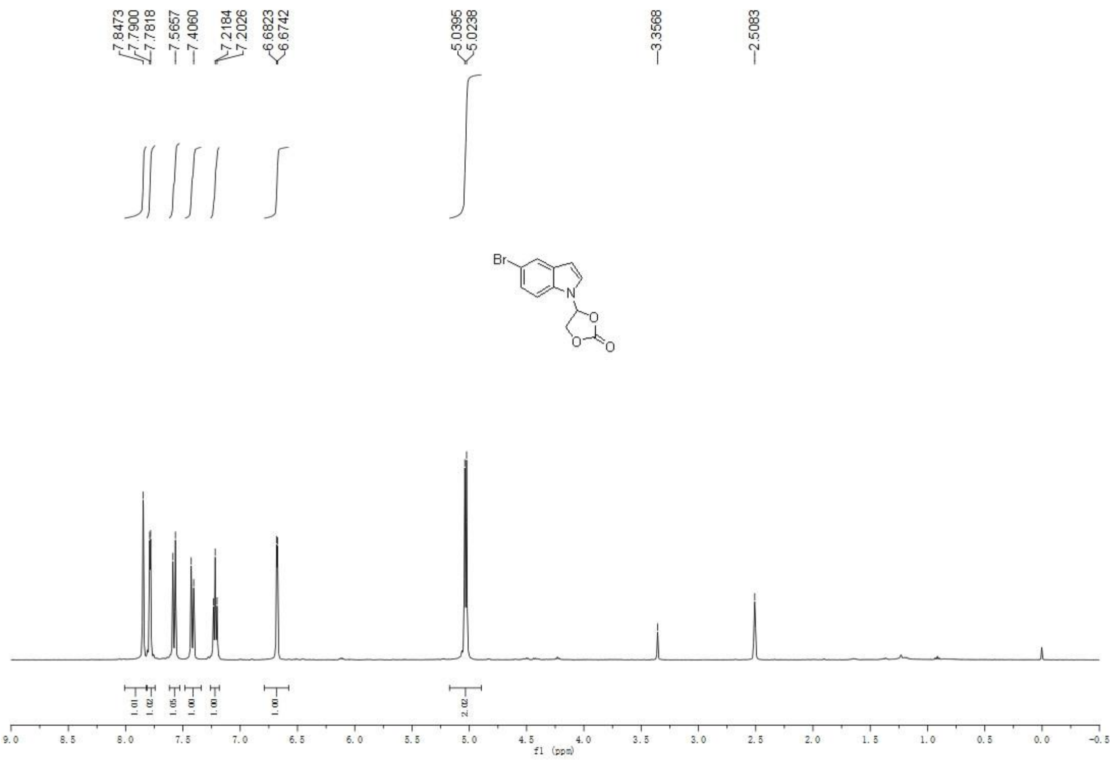

^{13}C NMR of 3r

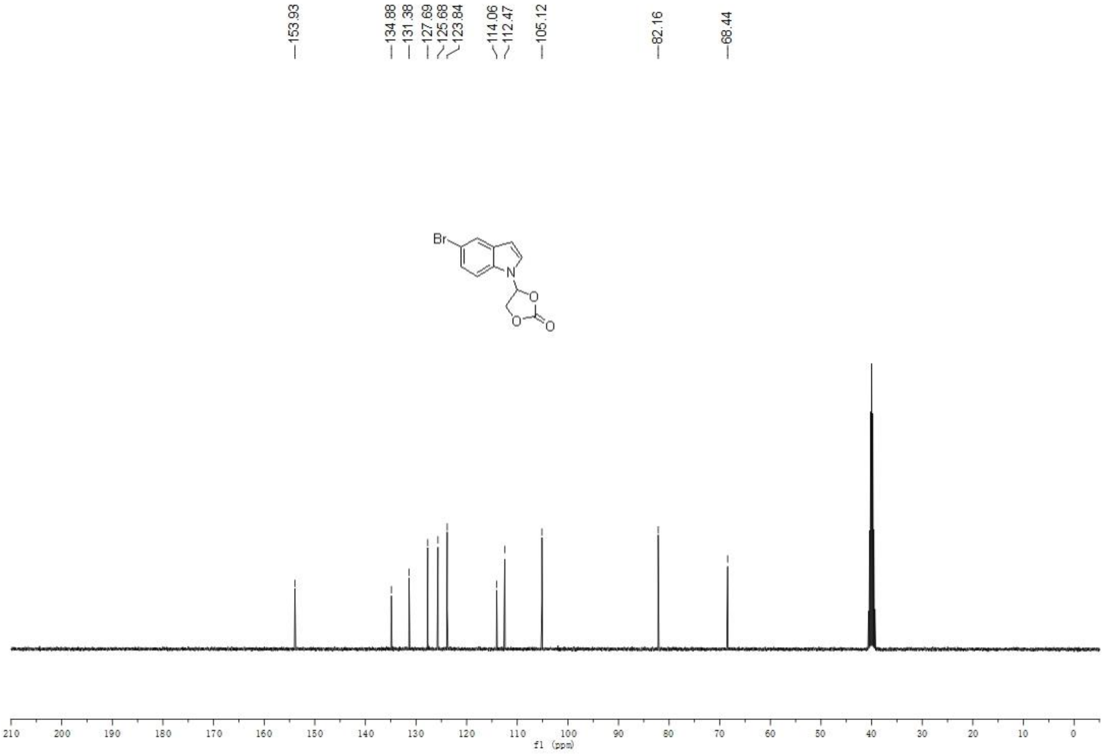

^{1}H NMR of 3s

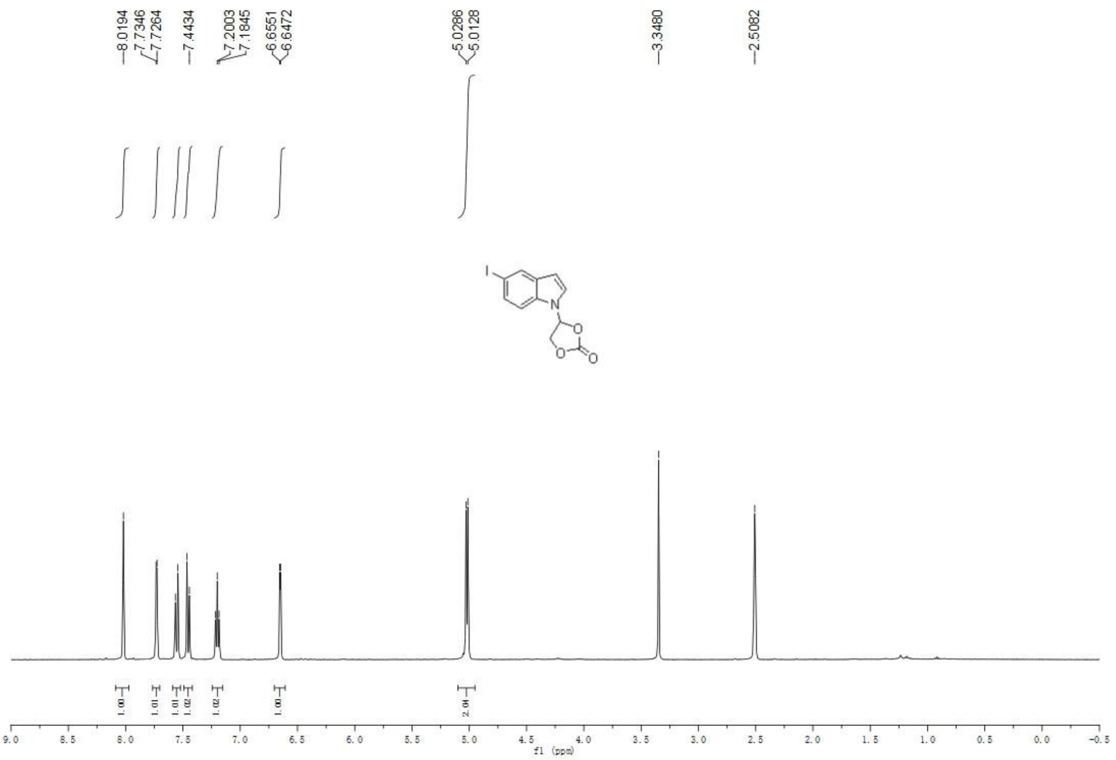

^{13}C NMR of 3s

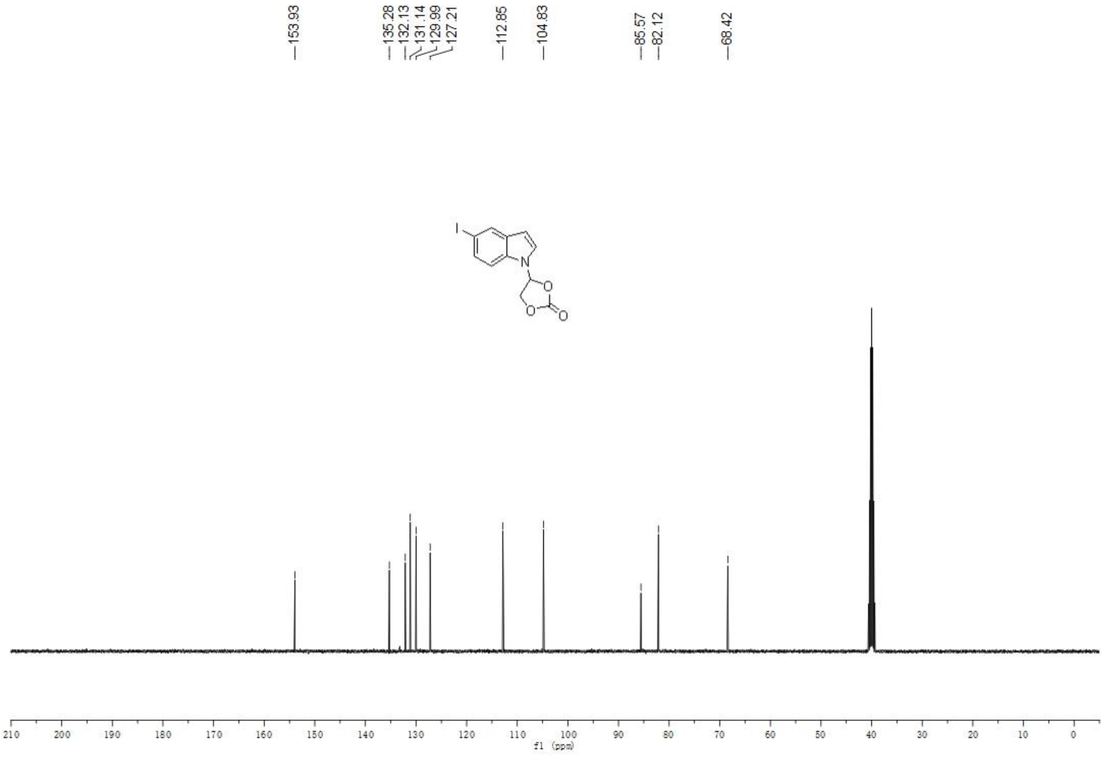

^{1}H NMR of 3t

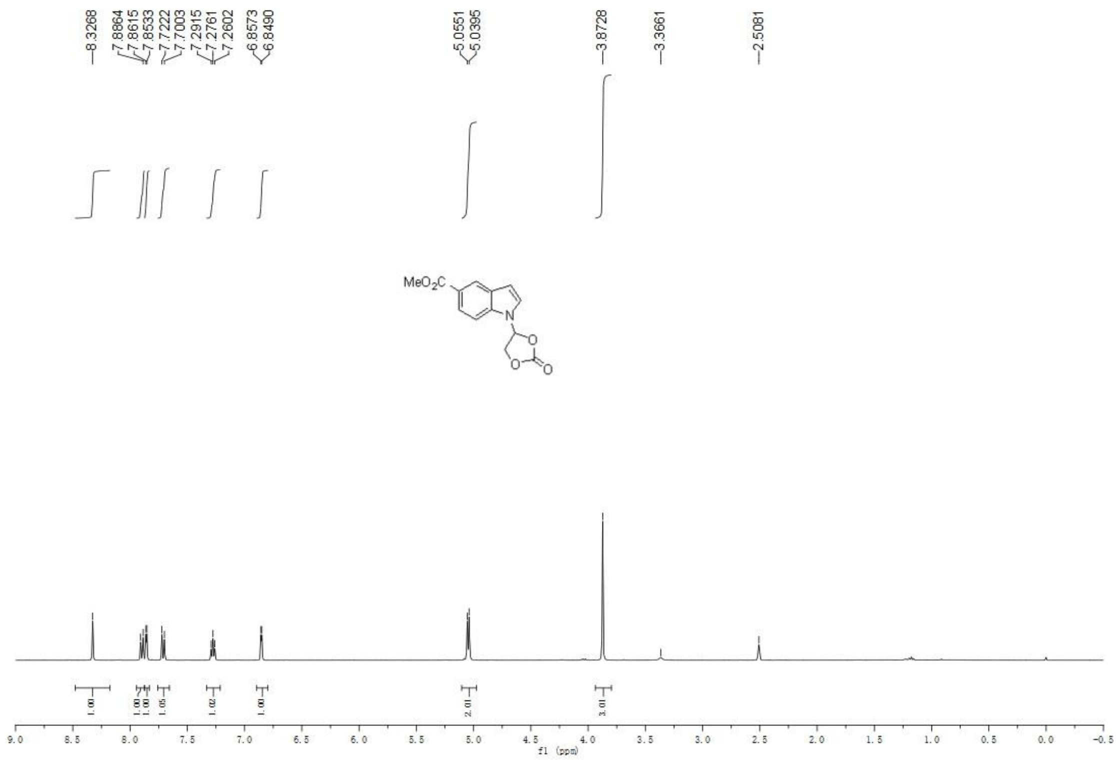

^{13}C NMR of 3t

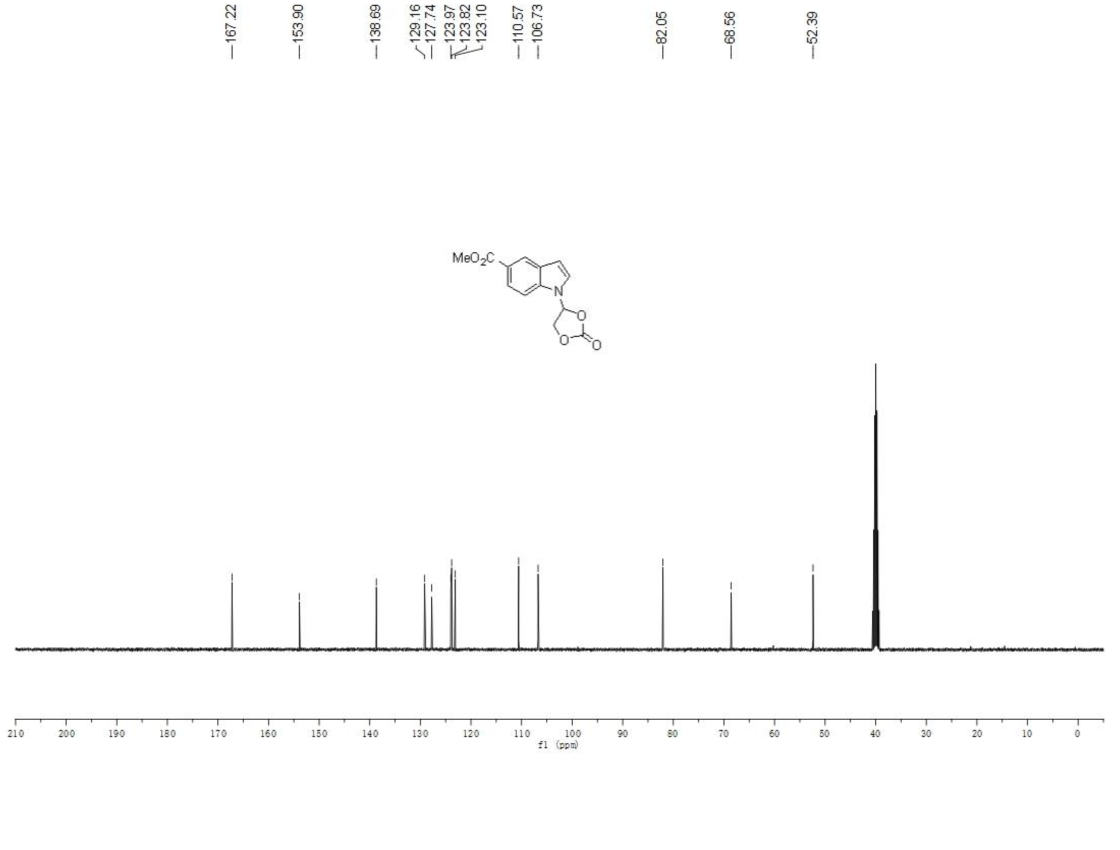

^{1}H NMR of 3u

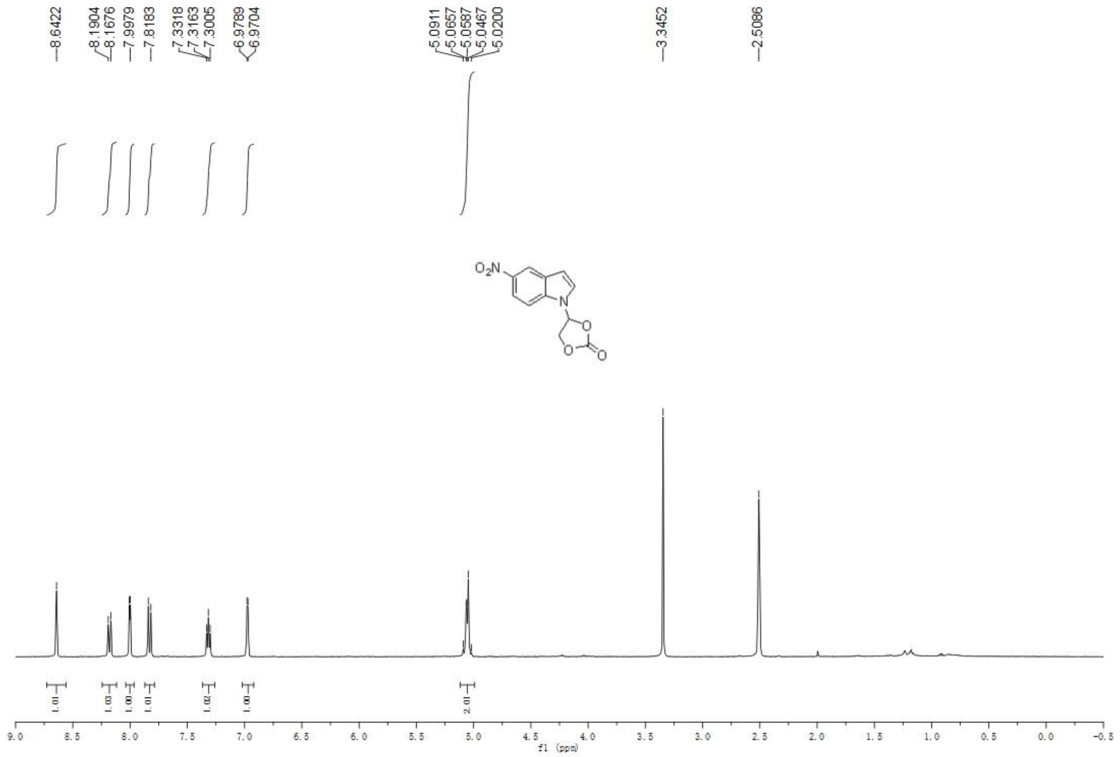

^{13}C NMR of 3u

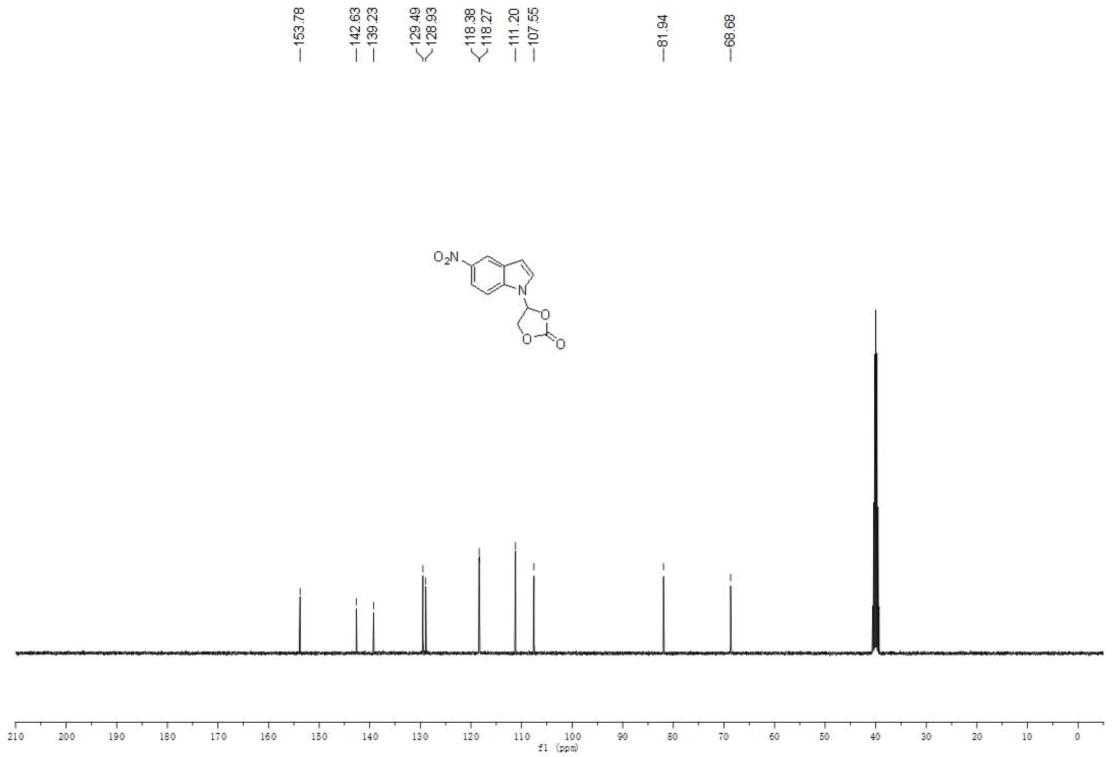

^{1}H NMR of 3v

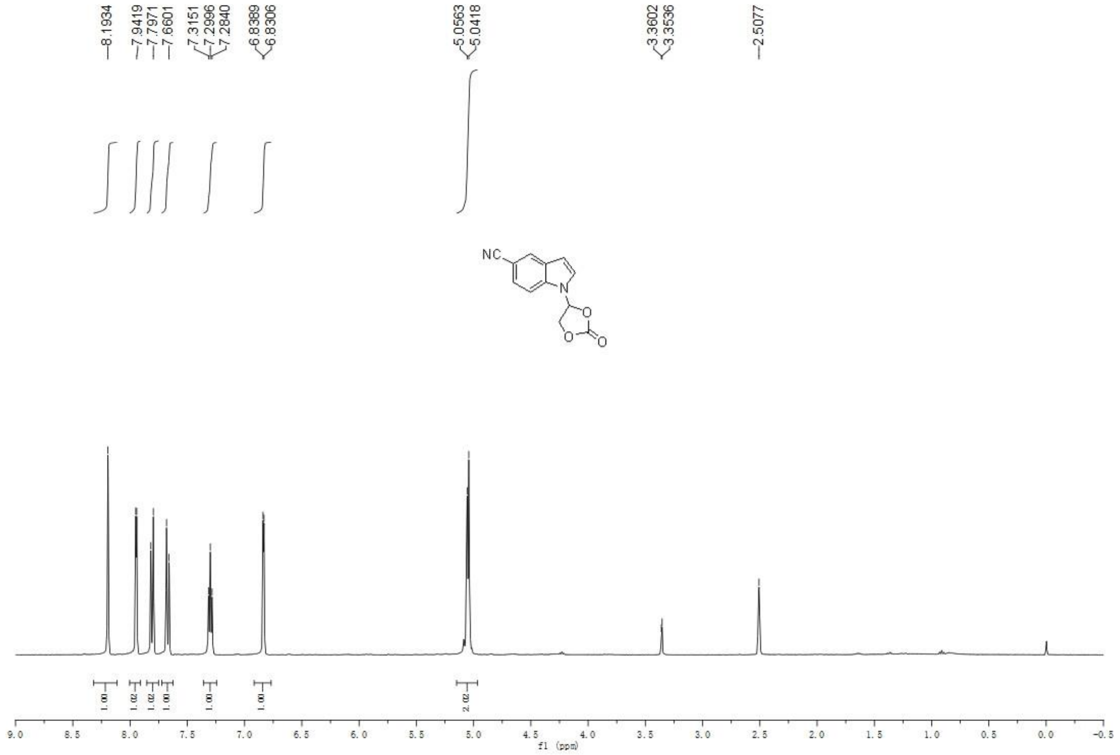

^{13}C NMR of 3v

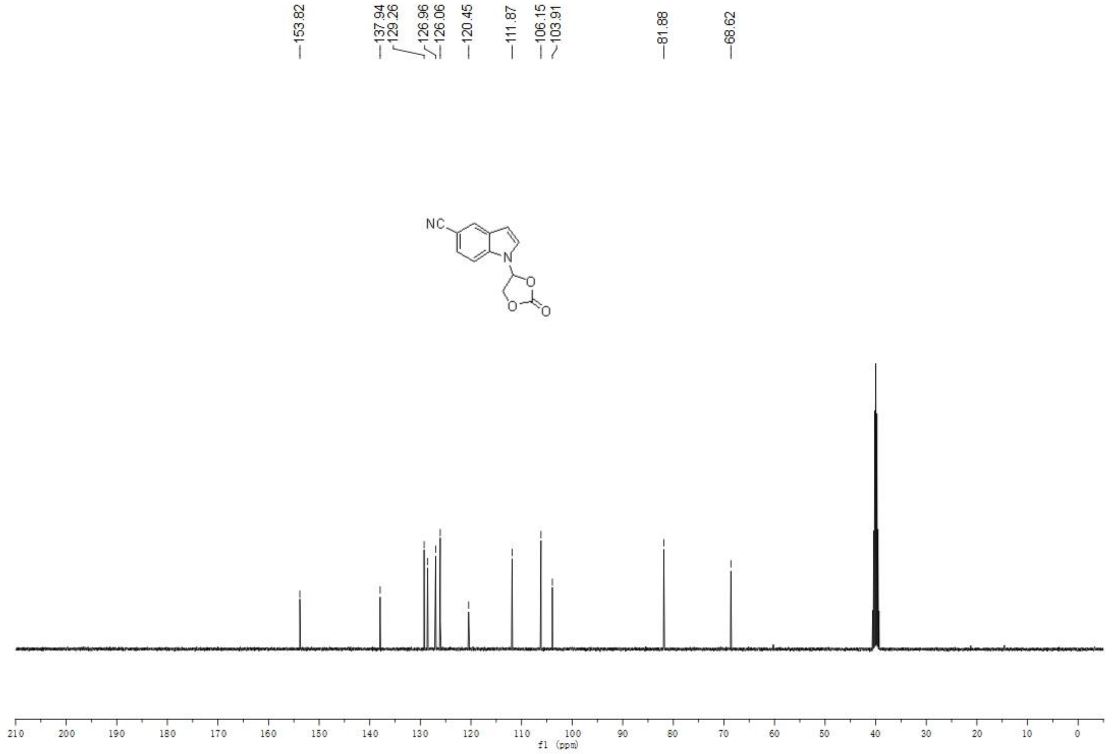

^{1}H NMR of 3w

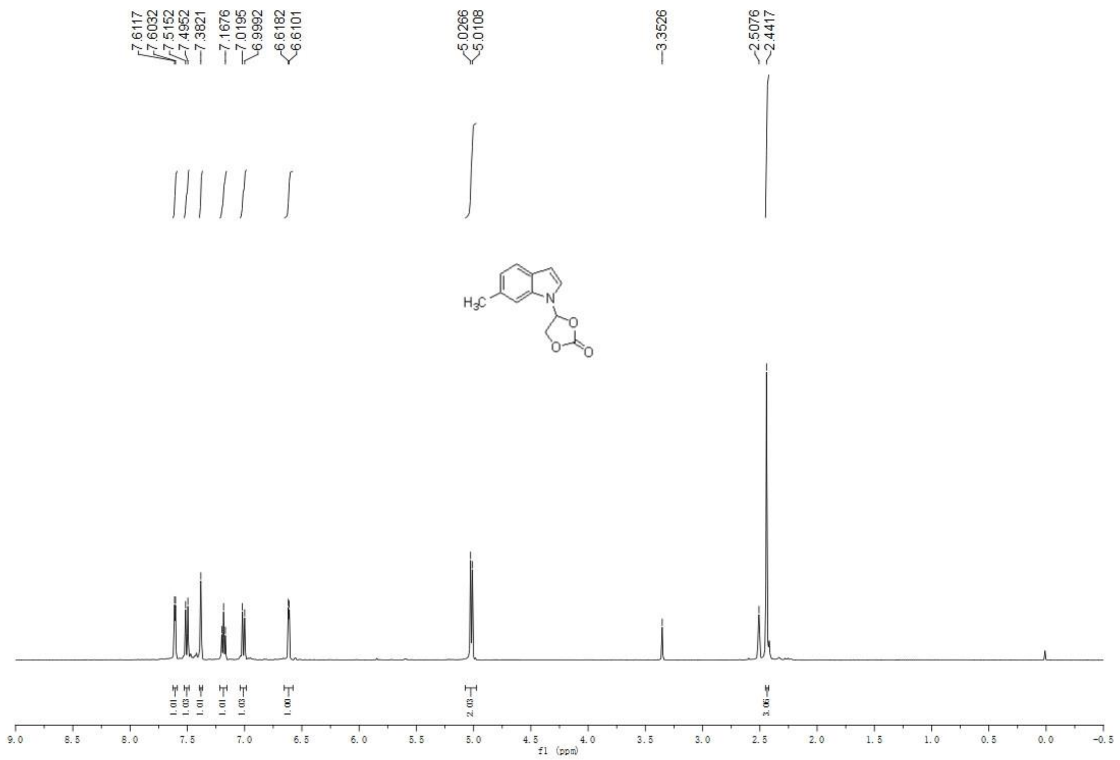

^{13}C NMR of 3w

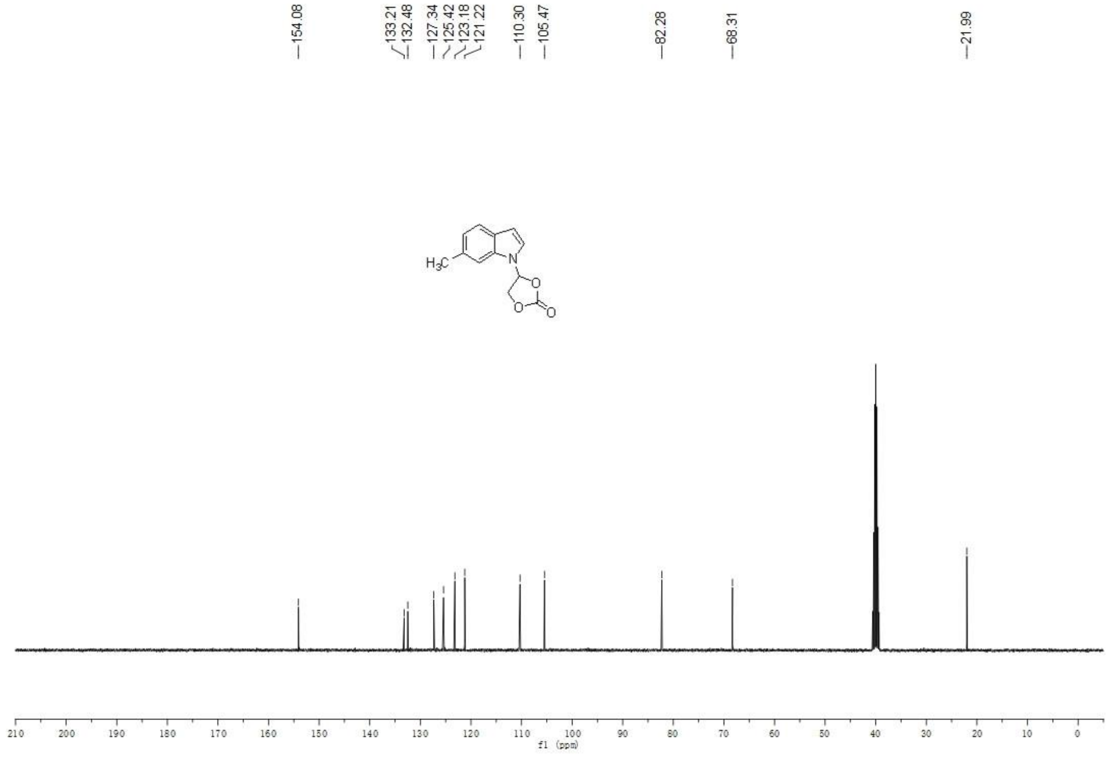

^{1}H NMR of 3x

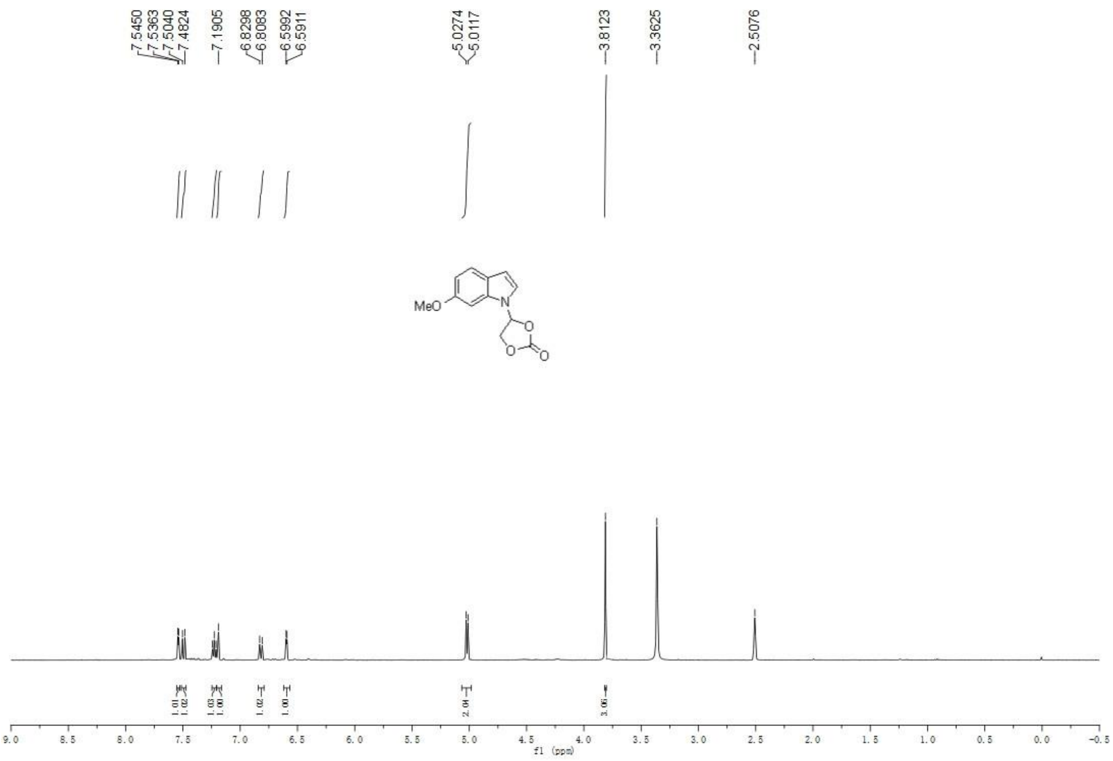

^{13}C NMR of 3x

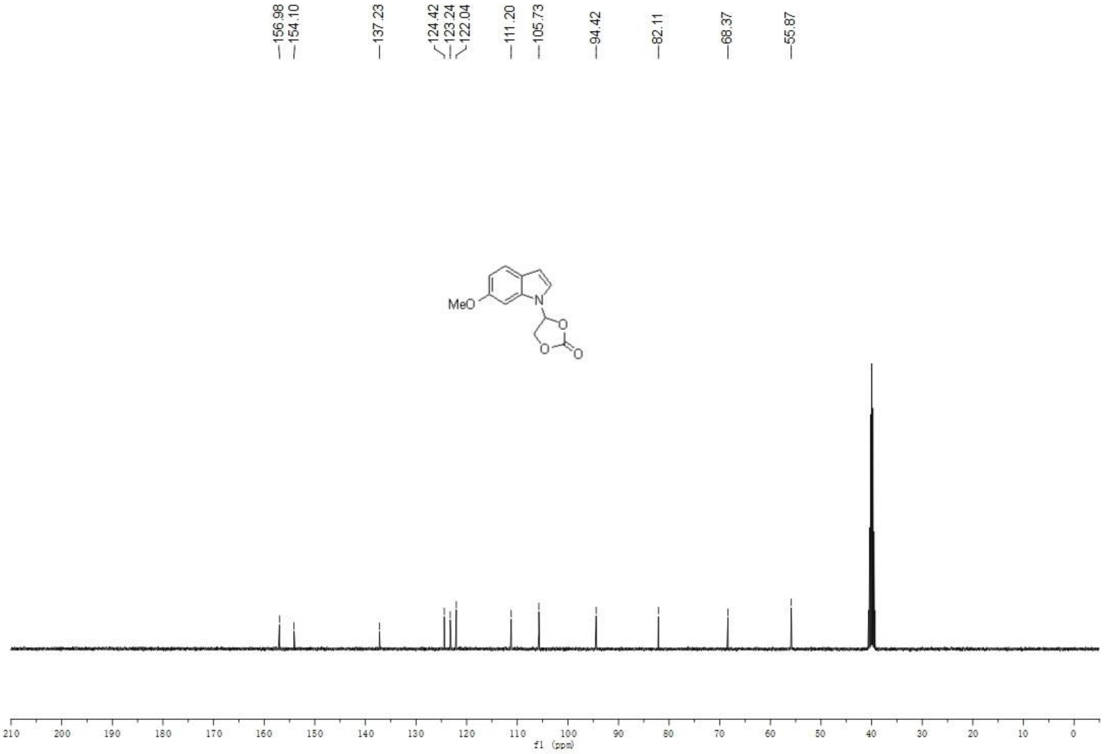

^{1}H NMR of 3y

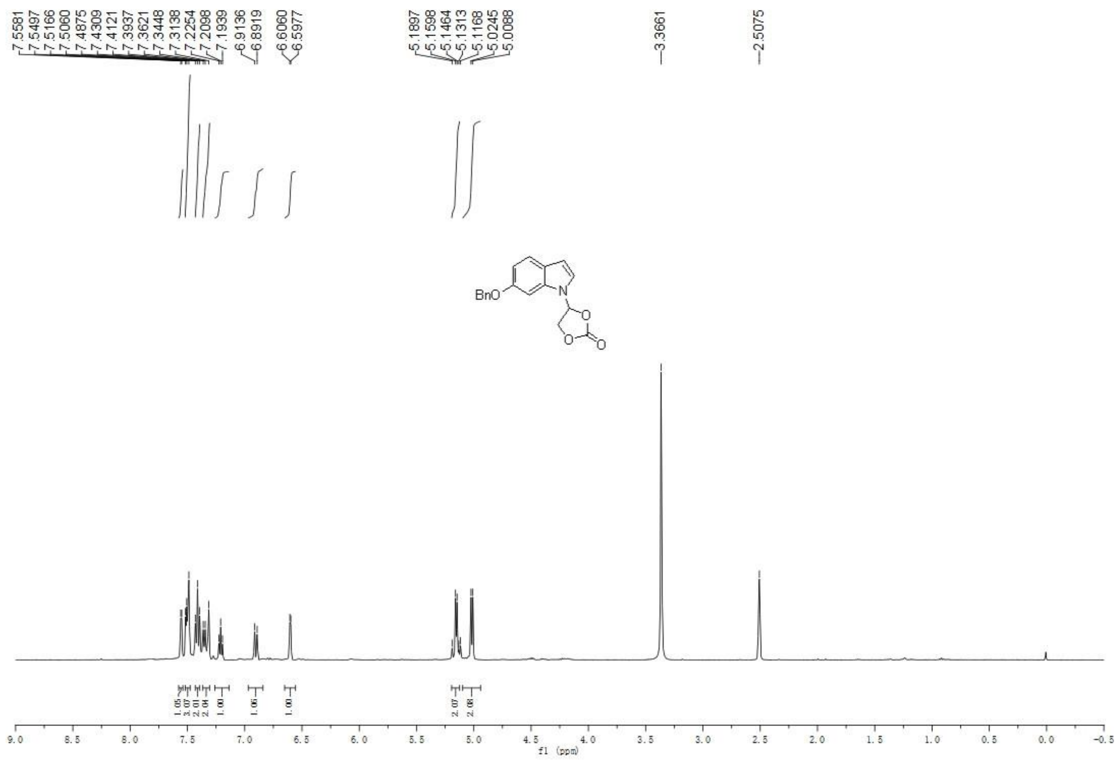

^{13}C NMR of 3y

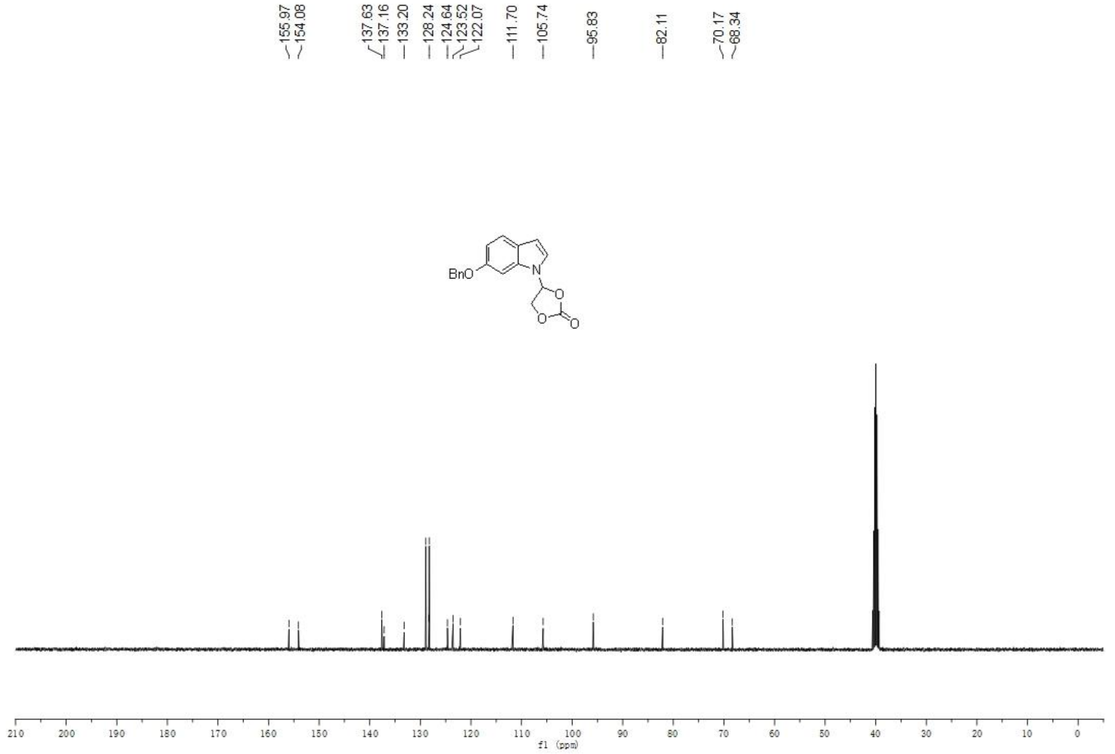

^{1}H NMR of 3z

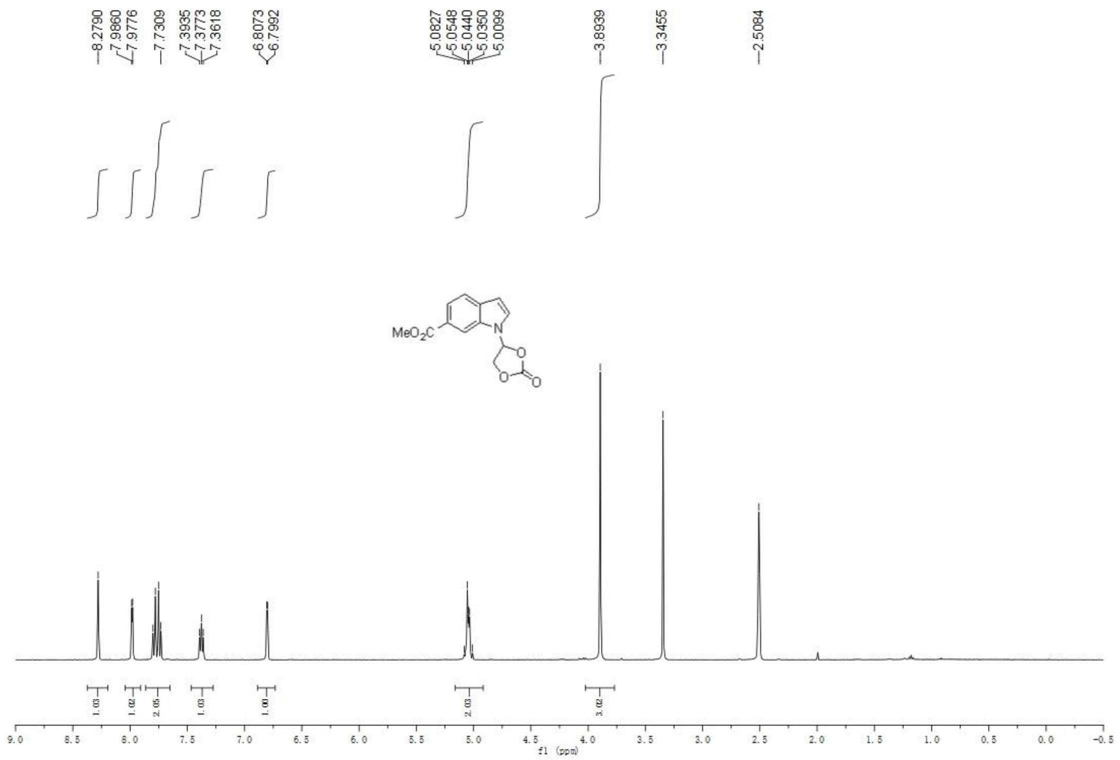

^{13}C NMR of 3z

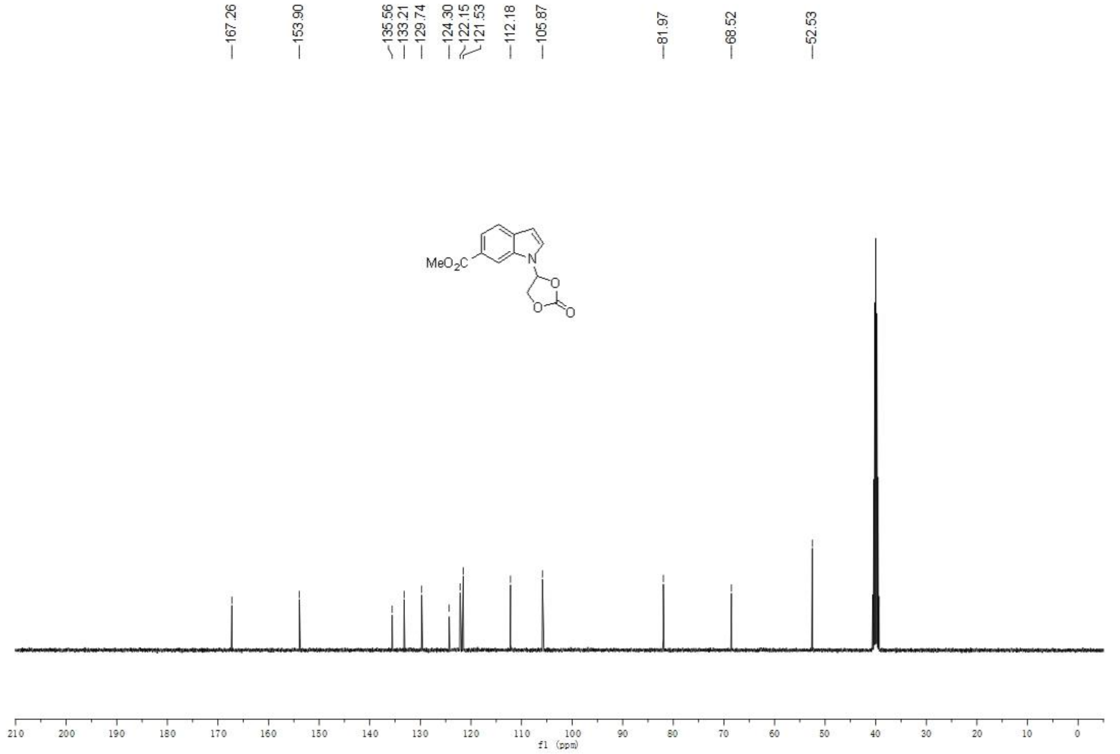

^{1}H NMR of 3aa

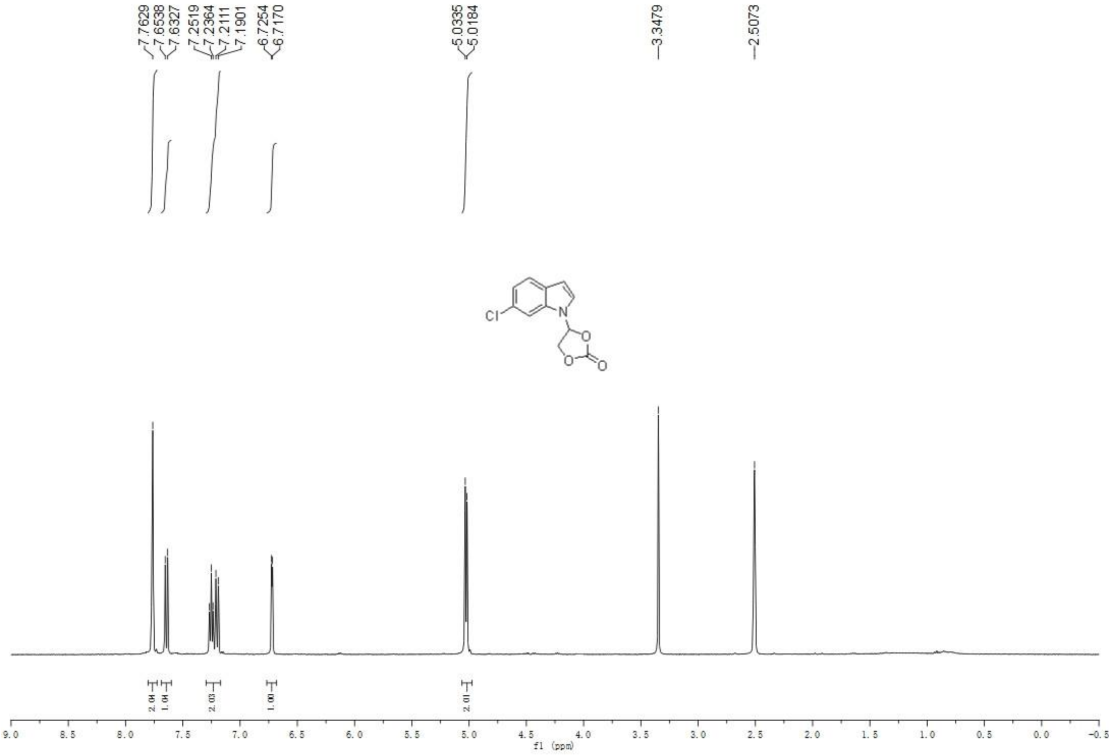

^{13}C NMR of 3aa

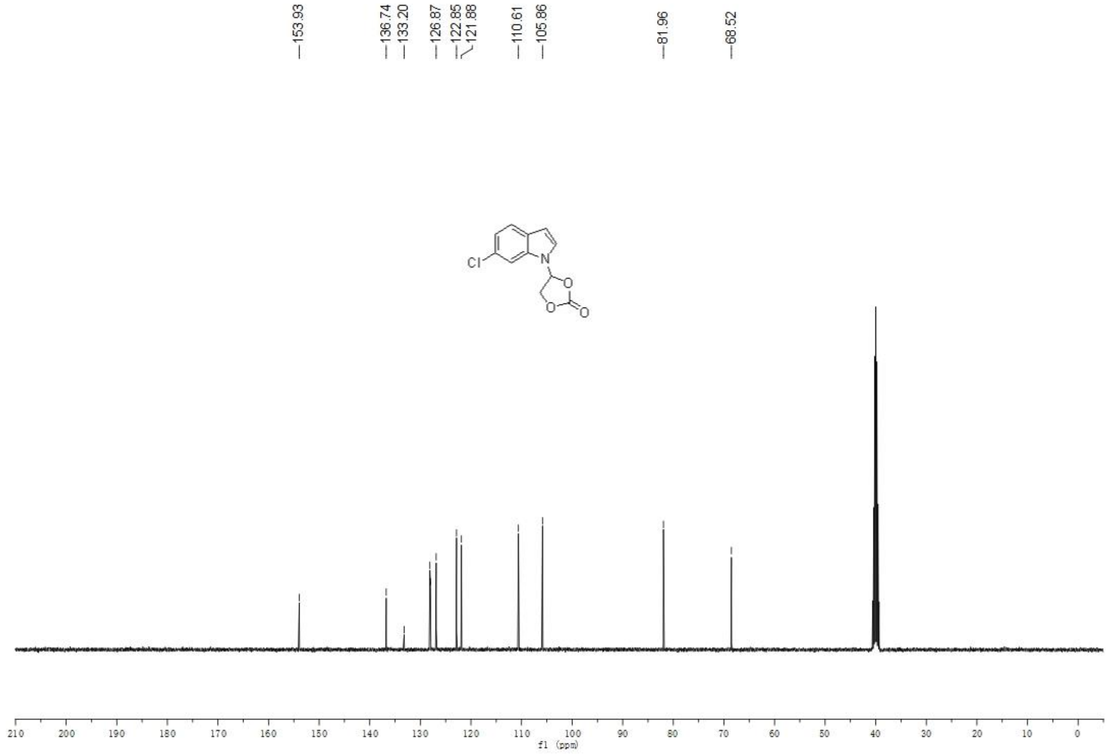

^{1}H NMR of 5

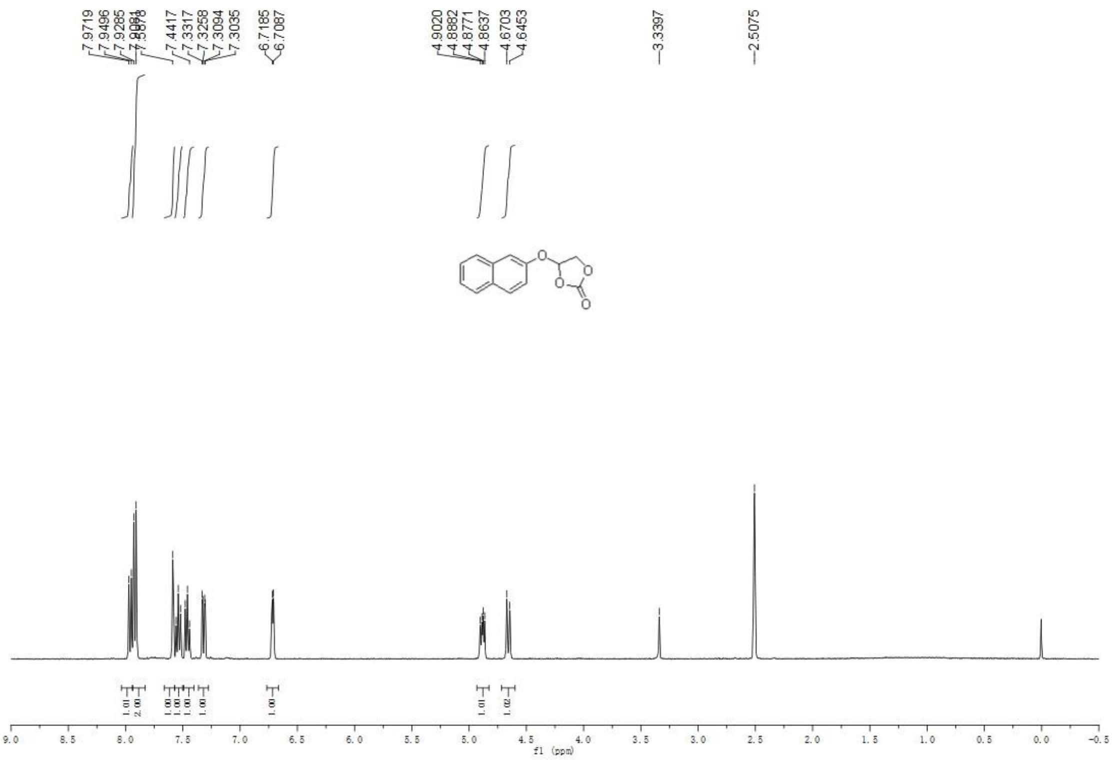

^{13}C NMR of 5

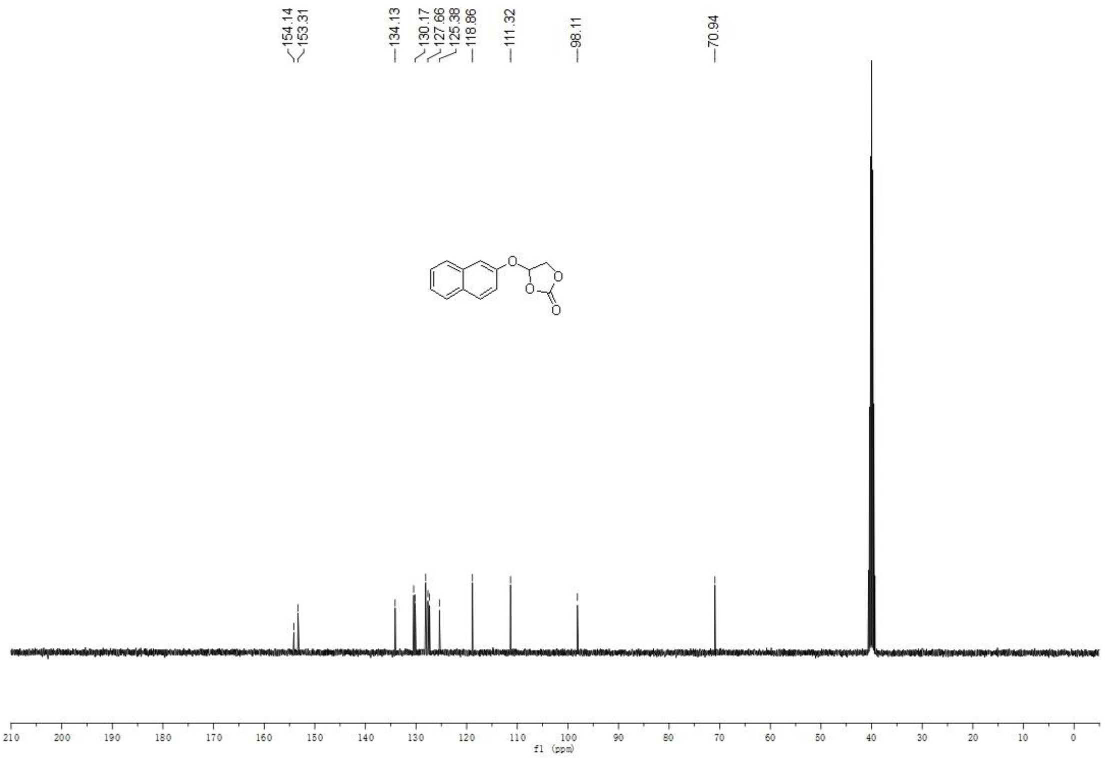

^{1}H NMR of 7

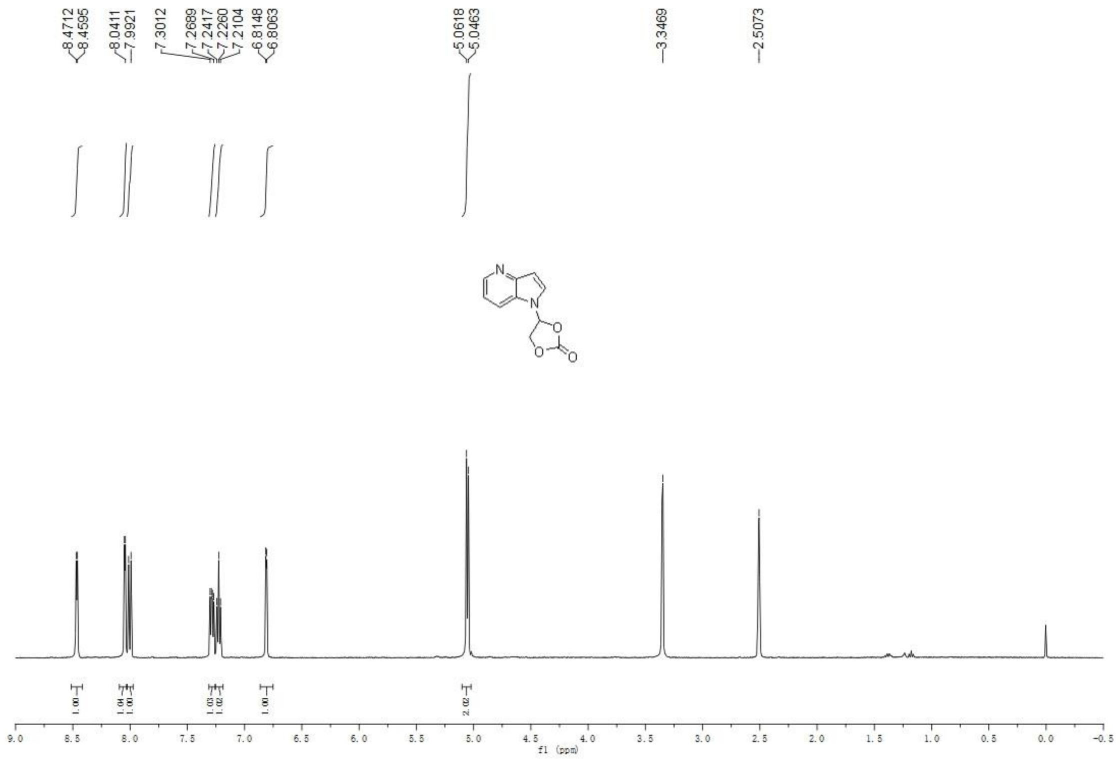

^{13}C NMR of 7

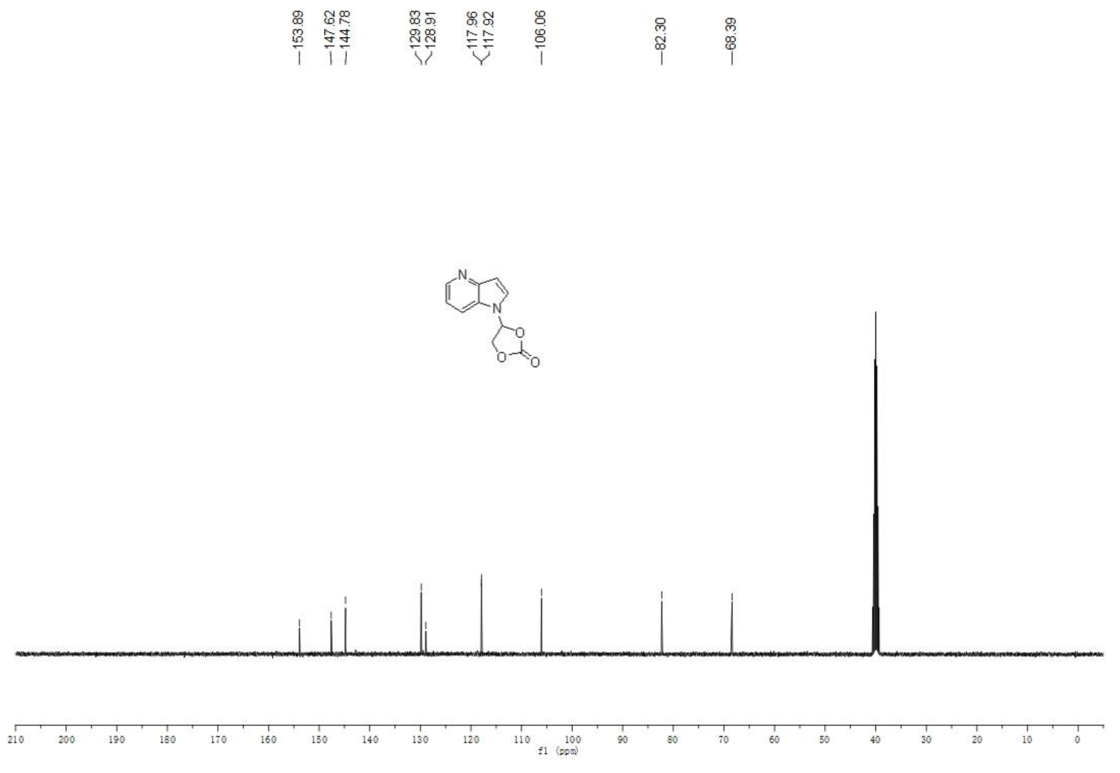

^{1}H NMR of 9

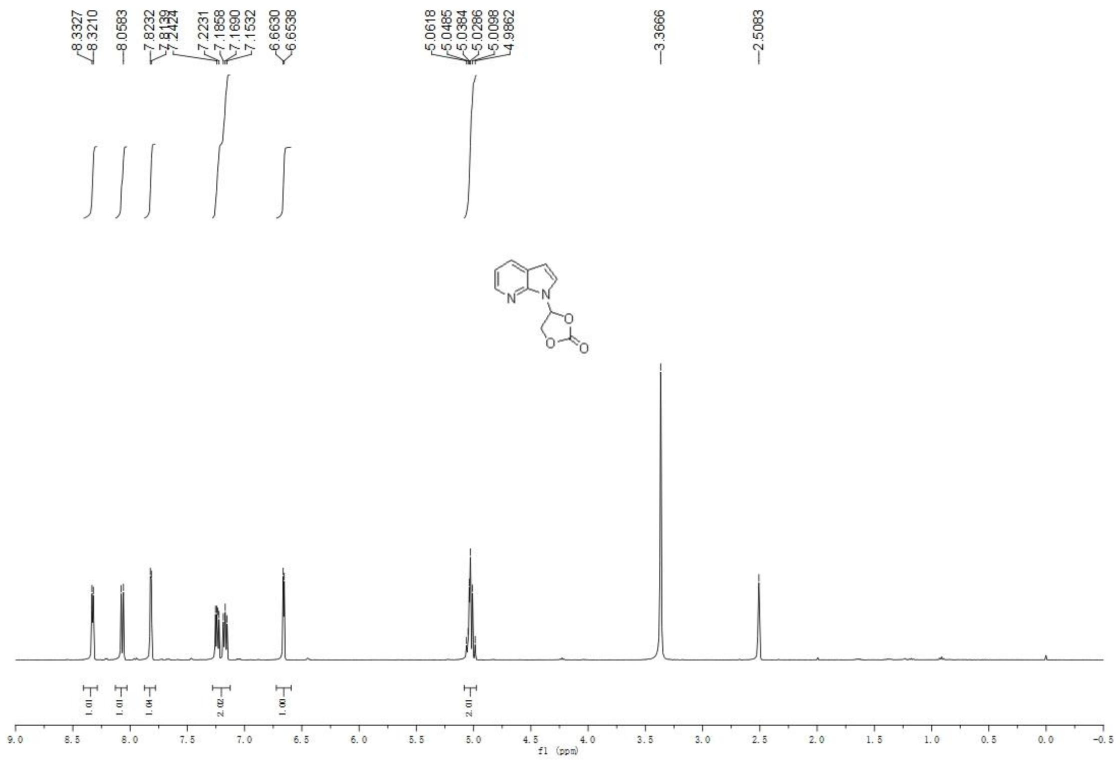

^{13}C NMR of 9

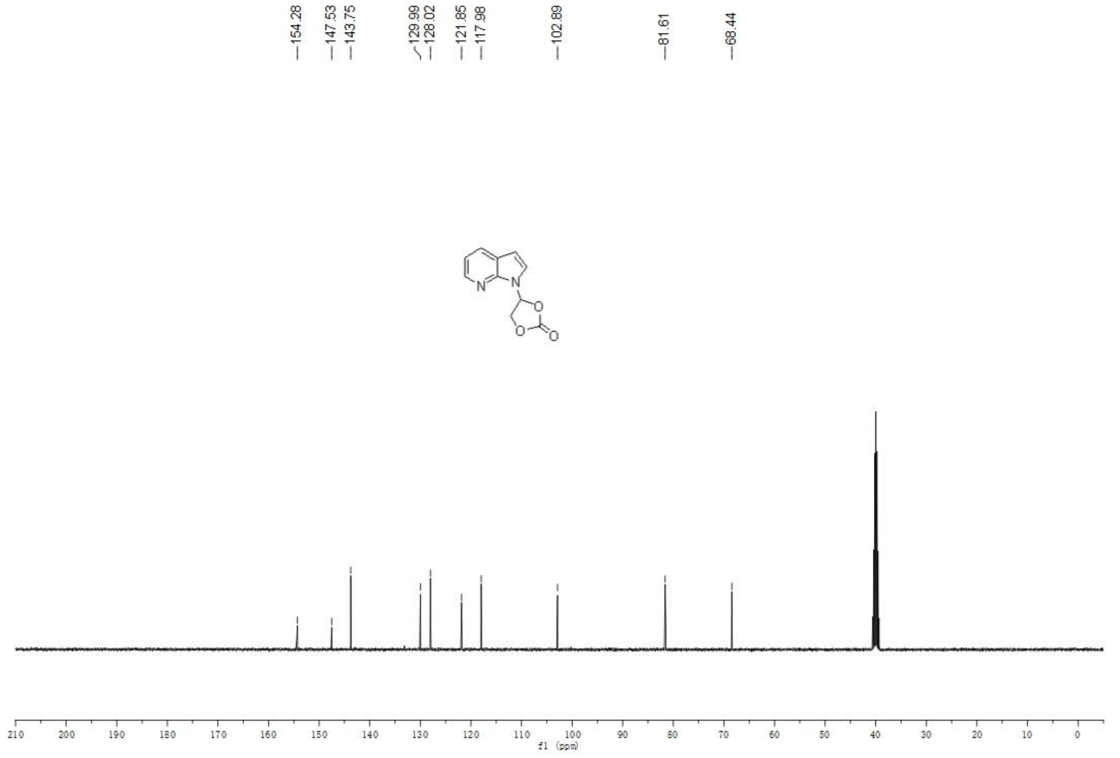

Supplement: Supplementary file 1 [file molecules-28-07450-s001.zip › SI-001-revised.pdf]
